# Bayesian adaptive design for pediatric clinical trials incorporating a community of prior beliefs

**DOI:** 10.1186/s12874-022-01569-x

**Published:** 2022-04-21

**Authors:** Yu Wang, James Travis, Byron Gajewski

**Affiliations:** 1 Department of Biostatistics & Data Science, University of Kansas Medical Center, Robinson 5028, 3901 Rainbow Blvd., Kansas City, KS 66160 USA; 2 Division of Biometrics II, Office of Biostatistics, Office of Translational Sciences, Center of Drug Evaluation and Research, U.S. Food and Drug Administration, Silver Spring, MD 20993 USA

**Keywords:** Bayesian adaptive design, Pediatric clinical trials, Prior belief, Interim analysis

## Abstract

**Background:**

Pediatric population presents several barriers for clinical trial design and analysis, including ethical constraints on the sample size and slow accrual rate. Bayesian adaptive design methods could be considered to address these challenges in pediatric clinical trials.

**Methods:**

We developed an innovative Bayesian adaptive design method and demonstrated the approach as a re-design of a published phase III pediatric trial. The innovative design used early success criteria based on skeptical prior and early futility criteria based on enthusiastic prior extrapolated from a historical adult trial, and the early and late stopping boundaries were calibrated to ensure a one-sided type I error of 2.5%. We also constructed several alternative designs which incorporated only one type of prior belief and the same stopping boundaries. To identify a preferred design, we compared operating characteristics including power, expected trial size and trial duration for all the candidate adaptive designs via simulation when performing an increasing number of equally spaced interim analyses.

**Results:**

When performing an increasing number of equally spaced interim analyses, the innovative Bayesian adaptive trial design incorporating both skeptical and enthusiastic priors at both interim and final analyses outperforms alternative designs which only consider one type of prior belief, because it allows more reduction in sample size and trial duration while still offering good trial design properties including controlled type I error rate and sufficient power.

**Conclusions:**

Designing a Bayesian adaptive pediatric trial with both skeptical and enthusiastic priors can be an efficient and robust approach for early trial stopping, thus potentially saving time and money for trial conduction.

**Supplementary Information:**

The online version contains supplementary material available at 10.1186/s12874-022-01569-x.

## Background

Children are often treated off-label due to the inadequacy or nonexistence of pediatric-specific safety and efficacy data [1, 2]. Meanwhile, the gap between adult approval and incorporation of pediatric information in drug labeling is substantial. For example, children tend to wait 6.5 years longer than adults to access new drugs on average [3]. Although clinical trials in children have resulted in significant improvements in their health care [4], the pediatric population inherently presents several barriers for clinical trial design and analysis, particularly, ethical constraints on sample sizes and prolonged recruitment processes. Ethical restrictions result from children’s status as a vulnerable population who “should not be enrolled in a clinical study unless necessary to achieve an important pediatric public health need” [5]. Difficulties also exist in the enrollment of pediatric patients because parents tend not to risk having their children exposed to unsure treatment effects [6, 7]. As a consequence of inadequate sample size or slow enrollment, pediatric clinical trials may be underpowered and yield inconclusive results [4]. Therefore, innovative methods such as adaptive designs are in demand to address these challenges and to identify effective treatments for the pediatric population in a timely manner.

Adaptive design methods have gained their popularity in the recent decade, and both the U.S. Food and Drug Administration (FDA) and the European Medicines Agency (EMA) have released guidance relating to their use. Adaptive design methods use the “learn as we go” approach which allows trials to adjust to information accumulated during the trial conduct that may not available when the trial began; therefore, they provide a variety of advantages over non-adaptive designs [8]. For example, adaptive design methods have the ability to stop a trial early if there is overwhelming evidence that the trial is unlikely to demonstrate efficacy at full accrual to reduce the number of patients exposed to ineffective drugs or stop a trial early if there is enough evidence that the trial would succeed to expedite patients’ access to efficacious medications.

Most traditional adaptive designs for clinical trials are based on frequentist methods, whilst in recent years Bayesian adaptive designs gained attention due to their flexibility of combining prior information with current information at the initial design stage, during the conduct of the trial, and at the analysis stage [9]. Also, it is easier to interpret adaptive trial designs using Bayesian methods than frequentist methods [10], and simulations can be used for Bayesian adaptive designs to evaluate the equivalent frequentist operating characteristics including power and type I error rate [11, 12].

Under the Bayesian framework, prior distribution refers to the probability distribution of our prior belief about the parameter of interest beforehand and the posterior distribution is our updated belief after seeing the data. Although the concept of applying Bayesian adaptive design methods has been widely discussed using noninformative prior with large variability for moderate and large clinical trials, noninformative prior may be problematic for pediatric clinical trials with small sample size as it can cause numerical instability and pathological posterior inference, and in order to obtain reliable inference, “the prior should be vague enough to cover the plausible values of the parameter, but not too vague to cause stability issues” [13, 14]. However, if a more informative prior could be justified, pediatric clinical trials are particularly well suited to benefit from Bayesian adaptive design methods. In practice, most pediatric studies are initiated after the same indication approved in adult population, therefore, a large amount of prior information exists for a new pediatric drug which has already been intensively tested on adults for safety and efficacy reasons [15]. Leveraging such prior information from historical adult trials can spare the need to start from scratch for testing a new treatment in pediatric patients under the assumption of sufficient similarity in disease progression and response to treatment between adult and pediatric studies [16, 17].

As first introduced by Kass and Greenhouse [18] and later summarized by Spiegelhalter [19], the idea of community of priors can be used to “describe a range of viewpoints that should be considered when interpreting evidence, and therefore a Bayesian analysis is best seen as providing a mapping from a space of specified prior beliefs to appropriate posterior beliefs” [21, p.160]. Recently, Ye, Reaman et al. [20] suggested that in a decision-making scenario for a pediatric clinical trial, models calculated under "skeptical” or”enthusiastic" prior beliefs can be considered simultaneously to control the type I error rate. Specifically speaking, historical adult study results showing treatment benefit could serve as an enthusiastic prior for futility criteria in the pediatric trial [20, 21], which allow us to stop a trial as soon as possible if the treatment effect is small or adverse despite the fact that we are enthusiastic that the treatment is efficacious, thereby minimizing exposure to ineffective medication for pediatric patients. Meanwhile, skeptical prior implying no treatment benefit also allows us to evaluate success criteria and stop the trial early when there is compelling efficacy evidence even though we are skeptical about the treatment benefit, so that pediatric patients could access to effective medication early.

In this paper, we applied an innovative Bayesian adaptive design method to a case study of a published phase III pediatric trial incorporating a community of prior beliefs. The early success criteria were based on skeptical prior while the early futility criteria were based on enthusiastic prior extrapolated from a historical adult trial. We also investigated the effect of an increasing number of interim analyses on the operating characteristics of the innovative design compared to several alternative designs incorporating only one prior belief to provide a recommendation on Bayesian adaptive design option for the case study.

## Methods

### Case study

The case study is a published phase III placebo-controlled randomized pediatric clinical trial to evaluate the safety and efficacy of a single treatment of two doses (4 U/kg and 8 U/kg) of Botox with standardized physical therapy (PT) in pediatric patients with lower limb spasticity on which pediatric approval was based. The same product was previously approved in adults on the basis of a single-phase III placebo-controlled study in a similar indication. In the pediatric trial, 412 subjects 2 to 16 years and 11 months of age were randomized in a 1:1:1 ratio to the Botox 8 U/kg group, Botox 4 U/kg group, or control group. The full label information is available at https://www.fda.gov/media/131444/download [22].

The original analyses for both the adult and pediatric trials were frequentist approaches, so we re-analyzed the primary efficacy endpoints using a Bayesian model to obtain posterior mean with standard deviation for the convenience of applying Bayesian adaptive design methods.

Table 1 summarizes both the pediatric and adult clinical trial designs and results of the primary efficacy endpoints used in the approval of Botox for the treatment of pediatric lower limb spasticity. For normal endpoint, the posterior distribution is approximately normal, so an approximate 95% credible interval (CI) can be computed as: posterior mean ± 2 × posterior SD. Then the approximate 95% CI for the treatment difference between Botox 4 U/kg group and control is (-0.10, 0.30) which contains zero, i.e., not enough evidence to declare treatment superiority to control. Therefore, we aimed at proposing an innovative Bayesian adaptive design to achieve treatment efficacy while maintaining good trial property.

**Table 1:** Comparison of adult and pediatric trial

	Historical Adult Trial	**Pediatric Trial**
Design	phase III, randomized, placebo-controlled	phase III, randomized, placebo-controlled
Trial Duration	1–2 years	4.5 years
Sample Size	Botox group (233) Ctrl. (235)	Botox 8 U/kg group (128) Botox 4 U/kg group (126) Ctrl. (130)
Primary Efficacy Endpoint Posterior mean (SD)	Botox group vs Ctrl.: 0.20 (0.10)	Botox 8 U/kg group vs Ctrl.: 0.27 (0.10) Botox 4 U/kg group vs Ctrl.: 0.10 (0.10)

*Trt* treatment, *Ctrl* control, *SD* standard deviation

### Prior beliefs

For the case study, we focused on the Bayesian analysis on two arms, the Botox 4 U/kg group and control group as the Botox 4 U/kg group was less efficacious (Table 1) and arm dropping is not the focus of our proposed method. We specified the priors separately for the two arms, which would lead to a prior on the difference between the Botox 4 U/kg treatment group and control group, and then we created a community of priors to be imposed on the difference between treatment (Botox 4 U/kg) and control to be consistent with the original analysis.

The skeptical prior is the pediatric stand-alone prior following a normal distribution with mean zero and standard deviation (SD) 0.5, which indicates no difference between treatment and placebo, i.e., skeptical viewpoint about treatment benefit. Our choice for standard deviation (SD) of the proposed skeptical prior was based on prior sensitivity analysis. We’ve investigated the impact of different choice of SD (0.1, 0.2, 0.5, 1, 2, 5, 10) on the posterior estimates of difference between treatment control and found that the posterior estimates were similar when SD ≥ 0.5. Therefore, we decided on a weakly-informative prior of $N({\mathrm{0,0.5}}^{2})$ for the difference between treatment and control. The enthusiastic prior is extrapolated from the adult trial results with mean 0.20 and SD 0.10 obtained from the adult trial posterior distribution, i.e., enthusiastic viewpoint about treatment benefit. The noninformative prior is a flat distribution with heavy tails centered at zero and SD 100, which provides no prior information with large variability and is therefore equivalent to frequentist approach, i.e., let the data speak for itself with no underlying strong opinion about treatment benefit. The choice of SD for noninformative prior was also based on sensitivity analysis. We also calculated prior effective sample size (ESS) to quantify the amount of information borrowed from the adult data through the prior [23]. We used variance-ratio (VR) method [24] to compute for prior ESS in our case of normal-normal model with conjugate prior. Based on Table 1, the variance of pediatric trial data is ${\sigma }^{2}={0.1}^{2}$, the prior ESS is $\frac{{\sigma }^{2}}{{\sigma }_{skep}^{2}}=\frac{{0.1}^{2}}{{0.5}^{2}}\approx 0.04$ for the skeptical prior and $\frac{{\sigma }^{2}}{{\sigma }_{enthus}^{2}}=\frac{{0.1}^{2}}{{0.1}^{2}}=1$ for the enthusiastic prior. Therefore, both the skeptical and enthusiastic prior have minimal informativeness. Additionally, the prior ESS is $\frac{{\sigma }^{2}}{{\sigma }_{noninf}^{2}}=\frac{{0.1}^{2}}{{100}^{2}}\approx 0$.000001 for the non-informative prior.

**Fig. 1:**
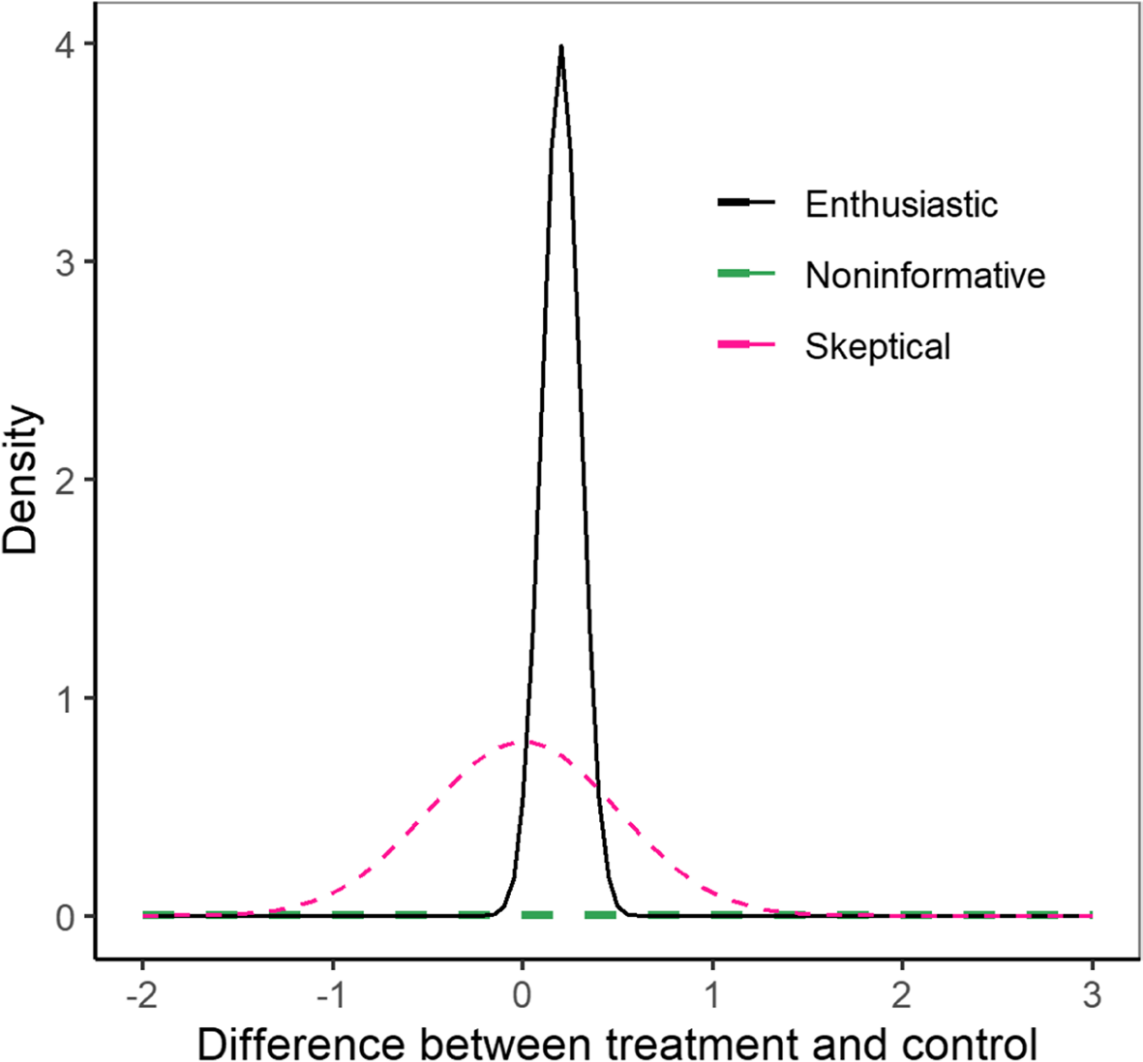
Distribution of Prior Beliefs

Figure 1 plots the distributions of these three different prior beliefs: the pink dashed line is the skeptical prior, the black solid line is the enthusiastic prior, and the green dashed line is the noninformative or flat prior.

### Bayesian adaptive designs

In this section, we will re-design the phase III pediatric clinical trial to illustrate an innovative Bayesian adaptive design method incorporating two prior distributions which represent two extreme ends of prior beliefs: skeptical and enthusiastic. For demonstration purposes, we focused on the Bayesian sequential monitoring for the treatment difference between the Botox 4 U/kg group and control group in the virtual execution of the pediatric trial. So, we are re-designing a new trial that has two arms and randomization is 1:1 for allocation to control and treatment (the Botox 4 U/kg group).

Under the context of the re-design using the proposed Bayesian adaptive design method, the early stopping criteria for success was based on skeptical prior and the early stopping criteria for futility was based on enthusiastic prior. We adopted the Haybittle–Peto approach for the choice of early decision boundaries [25, 26], i.e., the same threshold at every interim analysis:

- stop early for success based on skeptical prior if posterior probability
  $$\Pr\left(\mathrm{treatment}>\mathrm{control}\vert\mathrm{data},\mathrm{skeptical}\;\mathrm{prior}\right)>s_e$$
- stop early for futility based on enthusiastic prior if posterior probability
  $$\Pr\;\left(\mathrm{treatment}>\mathrm{control}\vert\mathrm{data},\mathrm{enthusiastic}\;\mathrm{prior}\right)<f_e$$

Where the early success boundary ${s}_{e}$ is the early success boundary and ${f}_{e}$ is the early futility boundary. The success and futility criteria were also evaluated at the final analysis:

- achieve late success based on skeptical prior if posterior probability
  $$\Pr\left(\mathrm{treatment}>\mathrm{control}\vert\mathrm{data},\mathrm{skeptical}\;\mathrm{prior}\right)>s_l$$
- achieve late futility based on enthusiastic prior if posterior probability
  $$\Pr\left(\mathrm{treatment}>\mathrm{control}\vert\mathrm{data},\mathrm{enthusiastic}\;\mathrm{prior}\right)<f_l$$

If the trial does not achieve any of the early or late success/futility criteria, inconclusive results will be obtained. Inconclusive pediatric clinical trials need to fulfill post marketing requirements without getting subsequent trials. Therefore, definitive answer is important in pediatric as it would prevent the delayed or non-use of beneficial therapies [4].

Under the framework of Bayesian methodology, null and alternative hypotheses are defined as different scenarios under which we assess the performance of the simulated trials [27]. The null and alternative hypotheses are ${H}_{0}:\delta =0$ versus ${H}_{1}:\delta >0$, where $\delta$ is the difference between the true treatment effect for the Botox 4 U/kg group and control group. For all the adaptive designs, the following *Operating Characteristics* were evaluated:

1. **Type 1 error rate**: under the null hypothesis scenario (${H}_{0}:\delta =0$) of having no difference, the proportion of such simulations that falsely declared the treatment was superior to control, i.e., the total proportions of early and late success under ${H}_{0}$
2. **Power**: under a particular alternative hypothesis scenario (${H}_{1}:\delta ={\delta }_{\mathrm{target}}$), of having a target difference of 0.05 (i.e., the observed difference between Botox 4 U/kg group and control is 0.05), the proportion of such simulations that concluded that the treatment was superior to control, i.e., the total proportions of early and late success under ${H}_{1}$
3. **Futility rate:** the total proportions of early and late futility under ${H}_{0}$ or ${H}_{1}$ separately
4. **Mean number of subjects:** the average sample size across all the simulations under ${H}_{0}$ or ${H}_{1}$ separately
5. **Mean trial duration:** the average trial duration (in weeks) across all the simulations under ${H}_{0}$ or ${H}_{1}$ separately

We need to calibrate and justify the decision boundary for the proposed innovative Bayesian adaptive design by exploring the effect of these boundaries on the *Operating Characteristics*. When determining the Haybittle–Peto boundary using the frequentist approach, the same threshold for level of significance is chosen at every interim analysis, i.e., 0.001 for the interim analysis, and the final analysis is performed using a standard threshold of 2.5% for level of significance. When using the Bayesian approach, the trade-off between the strength of skepticism in the prior and the early success boundary allows for more flexible decision making in the trial relative to the Haybittle-Peto boundary, i.e., a relaxed Haybittle-Peto approach. More skepticism in the prior impacts the final analysis, whereas increasing the early decision threshold avoids some of this impact, possibly at the cost of a lower early stopping rate when favorable results are seen. We chose 99.8% as the early success boundary because it balanced these concerns and controlled for overall type I error rate. The early futility boundary ${f}_{e}$ was tuned as 70% to maintain power. At the final analysis, the late futility boundary ${f}_{l}$ was set to be more stringent as 85%.

In addition to the innovative design, we also investigated the fixed design and several alternative adaptive designs with variations in early stopping criteria (Table 2). We started with fixed design which did not include any interim analysis, then moved on to investigate adaptive design options. As a comparison to adaptive design 3, we also looked at similar designs which only incorporate one type of prior belief at interim analysis: Bayesian adaptive design 1 only stop early for success based on skeptical prior while adaptive design 2 only stop early for futility based on enthusiastic prior. Similar to adaptive design 3, adaptive design 4 includes both early success and early futility decision rules, but all based on non-informative prior.

**Table 2:** Bayesian fixed/adaptive designs investigated

	**Early success**	**Early futility**	**Late success**	**Late futility**
Bayesian Fixed Design	-	-	-	-
Bayesian Adaptive Design 1	Skeptical prior	-	Skeptical prior	Skeptical prior
Bayesian Adaptive Design 2	-	Enthusiastic prior	Enthusiastic prior	Enthusiastic prior
Bayesian Adaptive Design 3	Skeptical prior	Enthusiastic prior	Skeptical prior	Enthusiastic prior
Bayesian Adaptive Design 4	Non-informative prior	Non-informative prior	Non-informative prior	Non-informative prior

Frequentist group-sequential design (GSD) is often considered as the benchmark for comparison. To ascertain that the Bayesian adaptive design 4 with non-informative prior is comparable to the frequentist GSD, we rerun the simulation with frequentist decision rule chosen to form 1-to-1 correspondence to the respective Bayesian decision boundary under non-informative prior, and calculated *p*-value based on one-sided t-test at both interim and final analyses. The Bayesian and corresponding frequentist decision rule at interim analysis:

- stop early for success based on noninformative prior if posterior probability
  $$\Pr\left(\mathrm{treatment}>\mathrm{control}\vert\mathrm{data},\mathrm{noninformative}\;\mathrm{prior}\right)>99.8\%$$
  Comparable to frequentist one-sided t-test *p*-value < 0.002
- stop early for futility based on Noninformative prior if posterior probability
  $$\Pr\left(\mathrm{treatment}>\mathrm{control}\vert\mathrm{data},\mathrm{noninformative}\;\mathrm{prior}\right)<70\%$$
  Comparable to frequentist one-sided t-test *p*-value > 0.3
  The Bayesian and corresponding frequentist decision rule at the final analysis:
- achieve late success based on noninformative prior if posterior probability
  $$\Pr\left(\mathrm{treatment}>\mathrm{control}\vert\mathrm{data},\mathrm{noninformative}\;\mathrm{prior}\right)>97.5\%$$
  Comparable to frequentist one-sided t-test *p*-value < 0.025
- achieve late futility based on noninformative prior if posterior probability
  $$\Pr\left(\mathrm{treatment}>\mathrm{control}\vert\mathrm{data},\mathrm{noninformative}\;\mathrm{prior}\right)<85\%$$

Comparable to frequentist one-sided t-test *p*-value > 0.15

We could compare the operating characteristics of the frequentist GSD to Bayesian adaptive design 4 with non-informative prior.

### Simulation Settings

Design simulations were performed using the Fixed and Adaptive Clinical Trial Simulator (FACTS) version 6.3 [28]. As for the execution aspects of the simulated trial, the maximum sample size was set to be 256 and the accrual rate was simulated in FACTS using a mean of 2 subjects per week with no dropouts, according to the original trial property. Patients were randomized to two arms (control, Botox 4 U/kg treatment) with equal allocation (1:1) and their scheduled visit was 12 weeks after randomization. The primary endpoint is a continuous variable following a normal distribution; therefore, Bayesian independent dose model was used under the FACTS Core Design-Continuous module:

$$Y\sim N({\theta }_{d},{\sigma }^{2})$$

$${\theta }_{d}\sim N({\mu }_{d}, {v}_{d}^{2})$$

$${\sigma }^{2} \sim {\text{Invers e-Gamma}}\left(\alpha ,\beta \right)=\text{Scaled-inverse-chi-squared}\left(\frac{{\sigma }_{n}}{2},\frac{{\sigma }_{\mu }^{2}{\sigma }_{n}}{2}\right)$$

where $d=1$ denotes the control group, $d=2$ denotes the Botox 4 U/kg treatment group. As mentioned before, different prior beliefs will be imposed on the difference between treatment and control, i.e., ${\theta }_{2}-{\theta }_{1}$. In FACTS, prior for each experimental arm needs to be specified separately, so to achieve the same prior specification as denoted in Fig. 1, we could introduce priors for ${\theta }_{d}, d=1, 2$ as follows:

- Under skeptical prior belief: ${\theta }_{1}\sim N(0, {0.3536}^{2})$, ${\theta }_{2}\sim N(0,{0.3536}^{2})$, so that ${\theta }_{2}-{\theta }_{1}\sim N\left(0, {0.5}^{2}\right)$ since $\sqrt{{0.3536}^{2}+{0.3536}^{2}}=0.5.$
- Under the enthusiastic prior belief: ${\theta }_{1}\sim N(0, {0.0707}^{2}), {\theta }_{2}\sim N(0.2, {0.0707}^{2}),$ so that ${\theta }_{2}-{\theta }_{1}\sim N\left(0.2, {0.1}^{2}\right)$ since $\sqrt{{0.0707}^{2}+0.0707}=0.1$.
- Under the noninformative prior belief:${\theta }_{1}\sim N\left(0, {70.71}^{2} \right), {\theta }_{2} \sim N\left(0,{70.71}^{2}\right)$, so that ${\theta }_{2}-{\theta }_{1}\sim N(0, {100}^{2})$ since$\sqrt{{70.71}^{2}+{70.71}^{2}}=100$.

For the prior imposed on ${\sigma }^{2}$, the Inverse-Gamma distribution could be reparametrized as the Scaled-inverse-chi-squared distribution [29]:

$${\chi }^{-2}\left({\sigma }^{2}|{\sigma }_{n}, {\sigma }_{\mu }\right)=\frac{1}{\Gamma \left(\frac{{\sigma }_{n}}{2}\right)} {\left(\frac{{\sigma }_{\mu }^{2}{\sigma }_{n}}{2}\right)}^{\frac{{\sigma }_{n}}{2}}{\left({\sigma }^{2}\right)}^{-\frac{{\sigma }_{n}}{2}-1}\mathrm{exp}\left(-\frac{{\sigma }_{\mu }^{2}{\sigma }_{n}}{2{\sigma }^{2}}\right)$$

where the parameter ${\sigma }_{n}>0$ is the degree of freedom or weight, and ${\sigma }_{\mu }>0$ is the scale or central value. As denoted in Gelman et al. [29], the Scaled-inverse-chi-squared distribution provides the information equivalent to ${\sigma }_{n}$ observations with squared standard deviation ${\sigma }_{\mu }^{2}$, and increasing ${\sigma }_{n}$ corresponds to increasing the effective strength of the prior. As for prior choice, weakly informative prior instead of noninformative prior was considered since the resulting posterior distribution was highly sensitive to the choice of weight ${\sigma }_{n}$ and scale ${\sigma }_{\mu }$, and noninformative on the log scale may not work [30]. Prior sensitivity analysis was conducted to investigate the impact of different prior distribution of ${\sigma }^{2}$ (different combinations of weight ${\sigma }_{n}$ and scale ${\sigma }_{\mu }$) on type I error rate, and we chose ${\sigma }_{n}=1, {\sigma }_{\mu }=0.07$ to control for type I error at the nominal level of 2.5%.

Using the specified model, we then performed FACTS simulations under different hypothetical subject response scenarios presented in Table 3. To optimize the number of interims, we also simulated trials which had between 1 and 18 interim analyses that were evenly spaced by number of patients enrolled (Table 4). Note that scenario with 0 interim is corresponding to the fixed design, which works as a reference for each of the adaptive designs. For each adaptive design candidate, 10,000 virtual trials were simulated in FACTS under each hypothetical scenario and each specification of number of interims. These simulations allow us to evaluate *Operating characteristics* including type I error rate and power, as well as estimating expected trial duration and number of subjects enrolled when performing an increasing number of interim analyses.

**Table 3:** Virtual Subject Response Scenarios

Scenario	Ctrl Mean (SD)	Trt Mean (SD)	Difference between Trt. and Ctrl Mean (SD)
Trt. 0 (H0, no difference)	0 (0.07)	0 (0.07)	0 (0.10)
Trt. 0.02 (Small difference)	0 (0.07)	0.02 (0.07)	0.02 (0.10)
Trt. 0.05 (H1, target difference)	0 (0.07)	0.05 (0.07)	0.05 (0.10)
Trt. 0.08 (Large difference)	0 (0.07)	0.08 (0.07)	0.08 (0.10)
Trt. -0.05 (Harmful scenario)	0 (0.07)	-0.05 (0.07)	-0.05 (0.10)

*Trt* treatment, *Ctrl* control, *SD* standard deviation

**Table 4:** Different number of interims investigated

Number of interims	Timing of planned interims (number of patients enrolled)
0	NA (Full accrual up to 256, no interim analysis)
1	128
2	85 170
3	64 128 192
4	51 102 153 204
5	43 86 129 172 215
6	37 74 110 146 183 220
7	32 64 96 128 160 192 224
8	28 56 84 112 140 168 196 224
9	25 50 75 100 125 150 175 200 225
10	23 46 69 92 115 138 161 184 207 230
12	20 40 60 80 100 120 140 160 180 200 220 240
14	17 34 51 68 85 102 119 136 153 170 187 204 221 238
18	13 26 39 52 65 78 91 104 117 130 143 156 169 182 195 208 221 234

*Operating characteristics* could be directly obtained from FACTS for fixed design & Bayesian adaptive design 1, 2, 4. As for the proposed adaptive design 3, Additional handling was conducted using R [31] for the FACTS output generated under the FACTS Core Design-Continuous module, and figures were produced using the package ggplot2 following the steps below (The FACTS screen-cuts and R code were provided in supplementary file 1):

Step 1: Create a FACTS adaptive design with the skeptical prior and include the interims and the QOIs but do not implement any stopping criteria so all interims are evaluated, and every simulation runs to full accrual and final analysis, then output weeks files for every simulation.

Step 2: Create a new FACTS adaptive design and change the prior to the enthusiastic prior and re-simulate without adaptation by keeping the same random number seed and making no other changes so that exactly the same patient responses are simulated.

Step 3: Aggregate the weeks files for designs simulated of the same trials but with skeptical or enthusiastic prior from Step 1 & 2 separately.

Step 4: Load the 2 sets of aggregated weeks files into R and join them on the Sim and Scenario ID columns so we have posterior probabilities under either skeptical or enthusiastic prior at each interim.

Step 5: Analyze the joined data for each simulated trial to see which stops early for success on the skeptical prior at interims, which stops early for futility on the enthusiastic prior at interims, which makes no early stopping up to full accrual or reach inconclusive at final analysis.

## Results

### Null and alternative scenarios

**Fig. 2:**
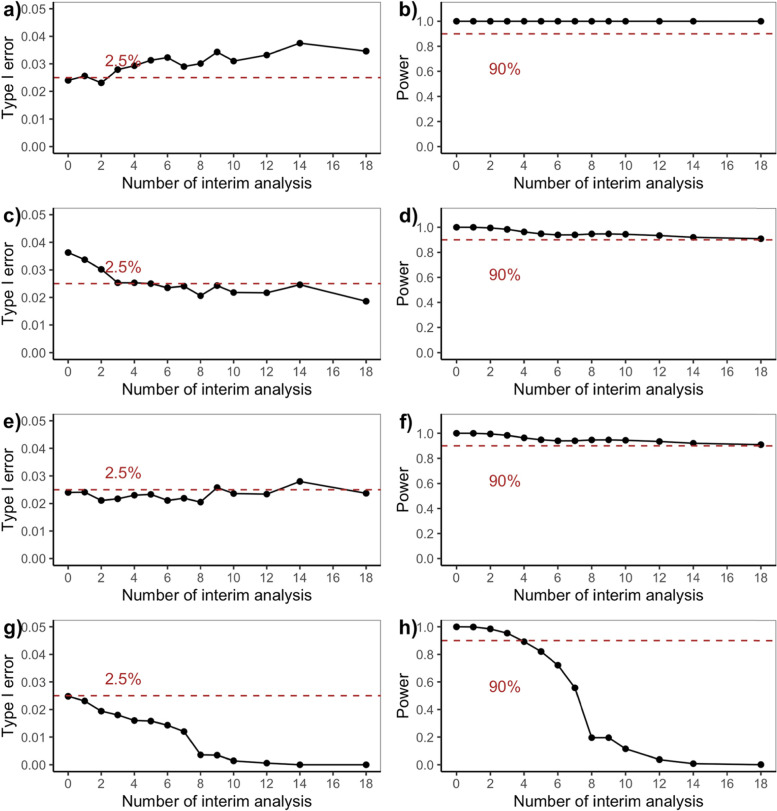
Type I Error (under H0) and Power (under H1) for Bayesian Adaptive Designs. **a** and (**b**) are Bayesian adaptive design 1 that only allow early stopping for success based on skeptical prior; (**c**) and (**d**) are Bayesian adaptive design 2 that only allow early stopping for futility based on enthusiastic prior; (**e**) and (**f**) are Bayesian adaptive design 3 that allow early stopping for success based on skeptical prior, or early stopping for futility based on enthusiastic prior; (**g**) and (**h**) are Bayesian adaptive design 4 that allow early stopping for either success or futility both based on non-informative prior

**Fig. 3:**
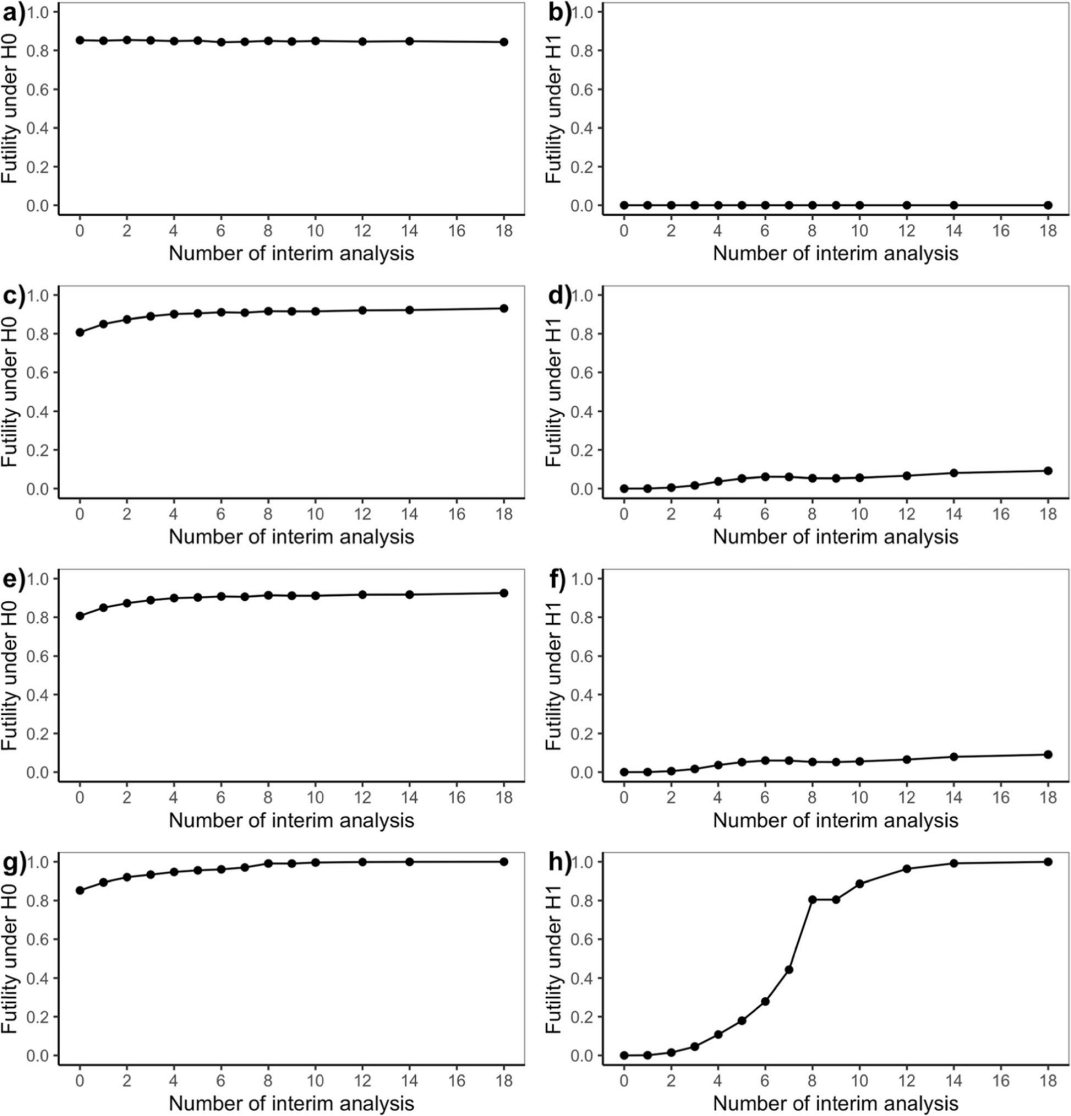
Futility Rate for Bayesian Adaptive Designs under Null (H0) and Alternative (H1) Scenarios. **a** and (**b**) are Bayesian adaptive design 1 that only allow early stopping for success based on skeptical prior; (**c**) and (**d**) are Bayesian adaptive design 2 that only allow early stopping for futility based on enthusiastic prior; (**e**) and (**f**) are Bayesian adaptive design 3 that allow early stopping for success based on skeptical prior, or early stopping for futility based on enthusiastic prior; (**g**) and (**h**) are Bayesian adaptive design 4 that allow early stopping for either success or futility both based on non-informative prior

**Fig. 4:**
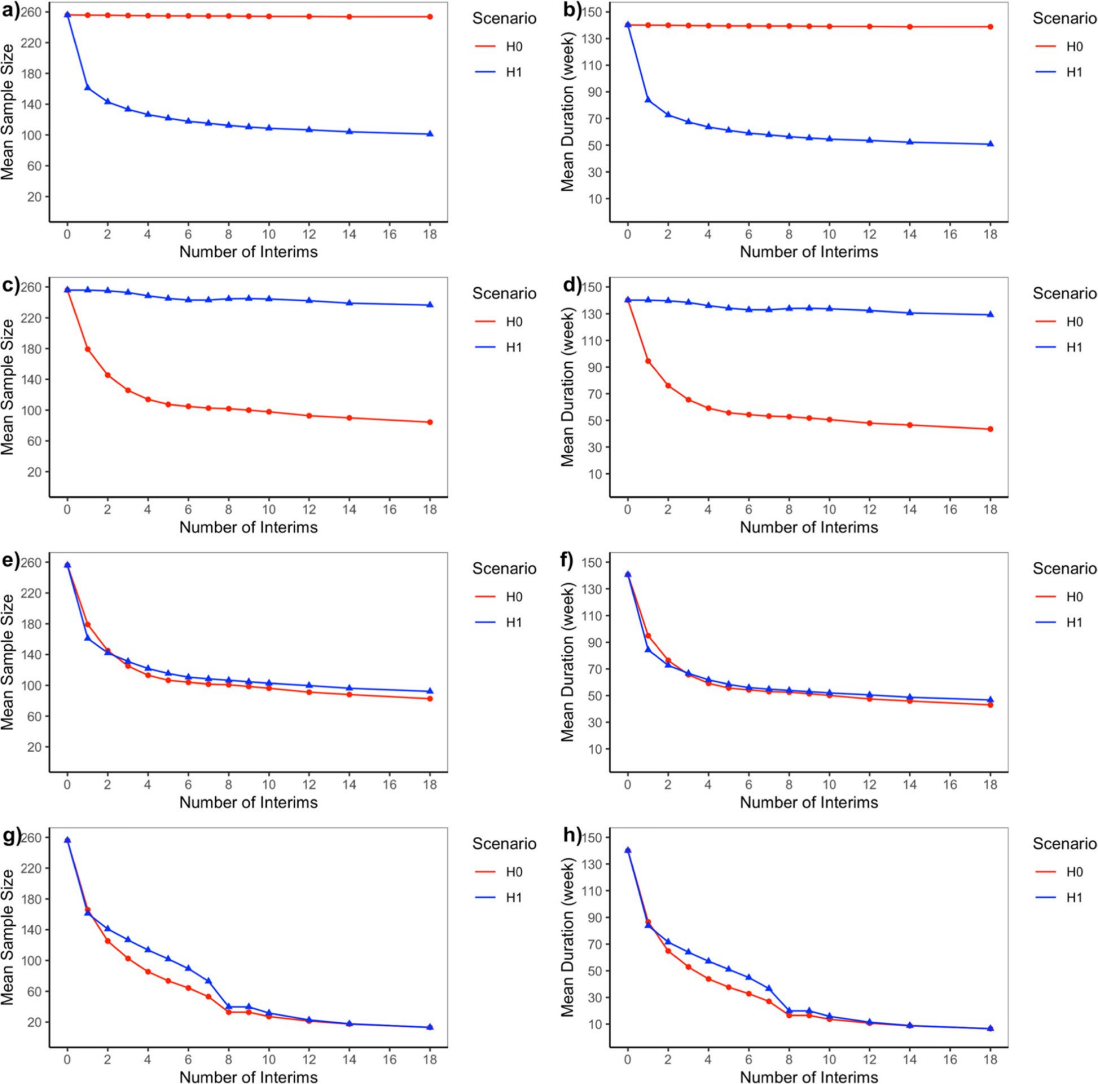
Mean Sample Size and Trial Duration for Bayesian Adaptive Designs under Null (H0) and Alternative (H1) Scenarios. **a** and (**b**) are Bayesian adaptive design 1 that only allow early stopping for success based on skeptical prior; (**c**) and (**d**) are Bayesian adaptive design 2 that only allow early stopping for futility based on enthusiastic prior; (**e**) and (**f**) are Bayesian adaptive design 3 that allow early stopping for success based on skeptical prior, or early stopping for futility based on enthusiastic prior; (**g**) and (**h**) are Bayesian adaptive design 4that allow early stopping for either success or futility both based on non-informative prior

As mentioned before, the null scenario is the case where there is no difference in treatment effects between Botox 4 U/kg group and control with an effect size of 0, and the alternative scenario is the case where the true treatment effect for Botox 4 U/kg group is superior to control with a target effect size of 0.5. The *operating characteristics* for Bayesian adaptive designs including type I error rate and power are presented in Fig. 2 while futility rate under the null or alternative scenarios are presented in Fig. 3. The expected sample size and trial duration are shown in Fig. 4. Note that the fixed design with no interim analysis (number of interim analysis = 0) works as a reference in each of the four adaptive design candidates.

In Fig. 2, the stopping boundaries for success or futility were adjusted to ensure the desired one-sided type I error of 2.5% for Bayesian adaptive design 3, and the same success or futility boundaries were used for designs 1, 2 and 4. Then we could compare type I error rate and power among all the adaptive design candidates as follows:

1. The type I error was first controlled but then gradually inflated (> 2.5%) with an overall increasing tendency, while the power was maintained (> 90%) without fluctuations when more interim analyses were included in the Bayesian adaptive design 1 that only allows early stopping for success based on skeptical prior (Fig. 2a and b).
2. The type I error was first inflated but then quickly controlled (< 2.5%) with a decreasing tendency in general, while the power was maintained (> 90%) with a slight drop when more interim analyses were included in the Bayesian adaptive design 2 that only allows early stopping for futility based on enthusiastic prior (Fig. 2c and 2d).
3. The type I error was generally increasing (< 2.5%) with small fluctuations, while the power was maintained (> 90%) with a slight decreasing trend [32] when more interim analyses were included in the Bayesian adaptive design 3 that allows early stopping for either success based on skeptical prior, or futility based on enthusiastic prior (Fig. 2e and 2f).
4. The type I error was generally controlled (< 2.5%) with a strong decreasing tendency, while the power was heavily affected (< 90%) and tends to zero when more interim analyses were included in the Bayesian adaptive design 4 that allows either early stopping for either success or futility both based on non-informative prior (Fig. 2g and 2h).

According to Fig. 2, Bayesian adaptive design 1 yields inflation of type I error rate, which requires stricter skeptical prior or success boundaries. In terms of power, the loser would be Bayesian adaptive design 4 since the power almost drops down to zero when performing an increasing number of interims although type I error rate decreases because of the trade-off between type I and type II error rate. Note that Bayesian adaptive design 4 incorporating non-informative prior corresponds to a frequentist Pocock design [12], which is often criticized for giving too high a probability of early stopping. The same story could be told in Fig. 3 where most Bayesian adaptive designs had futility rate under the alternative scenario controlled under 10% except for adaptive design 4 in which false futility was claimed so that power was affected. Figure 4 shows that the expected sample size is considerably reduced by many interim analyses for Bayesian adaptive design 3 under both the null and alternative scenarios.

To help explain the nuances, the operating characteristics for Bayesian adaptive design 1–4 are provided as Tables 1, 2, 3, and 4 in supplementary file 2, which combines information from Figs. 1 and 3 to facilitate the comparison between the proposed design and several alternatives (only mean sample size is presented as it behaves similarly to mean trial duration). The operating characteristics for frequentist GSD are provided as Table 5 in supplementary file 2. We could see that the operating characteristics of the frequentist design are comparable to Bayesian adaptive design 4 with non-informative prior, consistent with the findings in [12].

According to the operating characteristics presented so far, generally speaking, when an increasing number of interim analyses were performed, we could observe a slight decrease in power and a small inflation in type I error rate or futility rate. Also, as expected Bayesian adaptive design 3 is the best design since it produces the greatest reduction in sample size as well as trial duration while still controlling for type I error rate and maintaining sufficient power. Bayesian adaptive designs 1 & perform as one you expect—showing an inflated type I error rate. And the lack of futility analyses makes the trial continue to full accrual under the null scenario, while for Bayesian adaptive design 2 we see sufficient power and control of the type I error rate, but no reduction in sample size under the alternative scenario since there is no interim efficacy analysis. Bayesian adaptive design 4 aggressively minimizes sample size at a sacrifice of power making the design undesirable.

### Harmful scenario

**Fig. 5:**
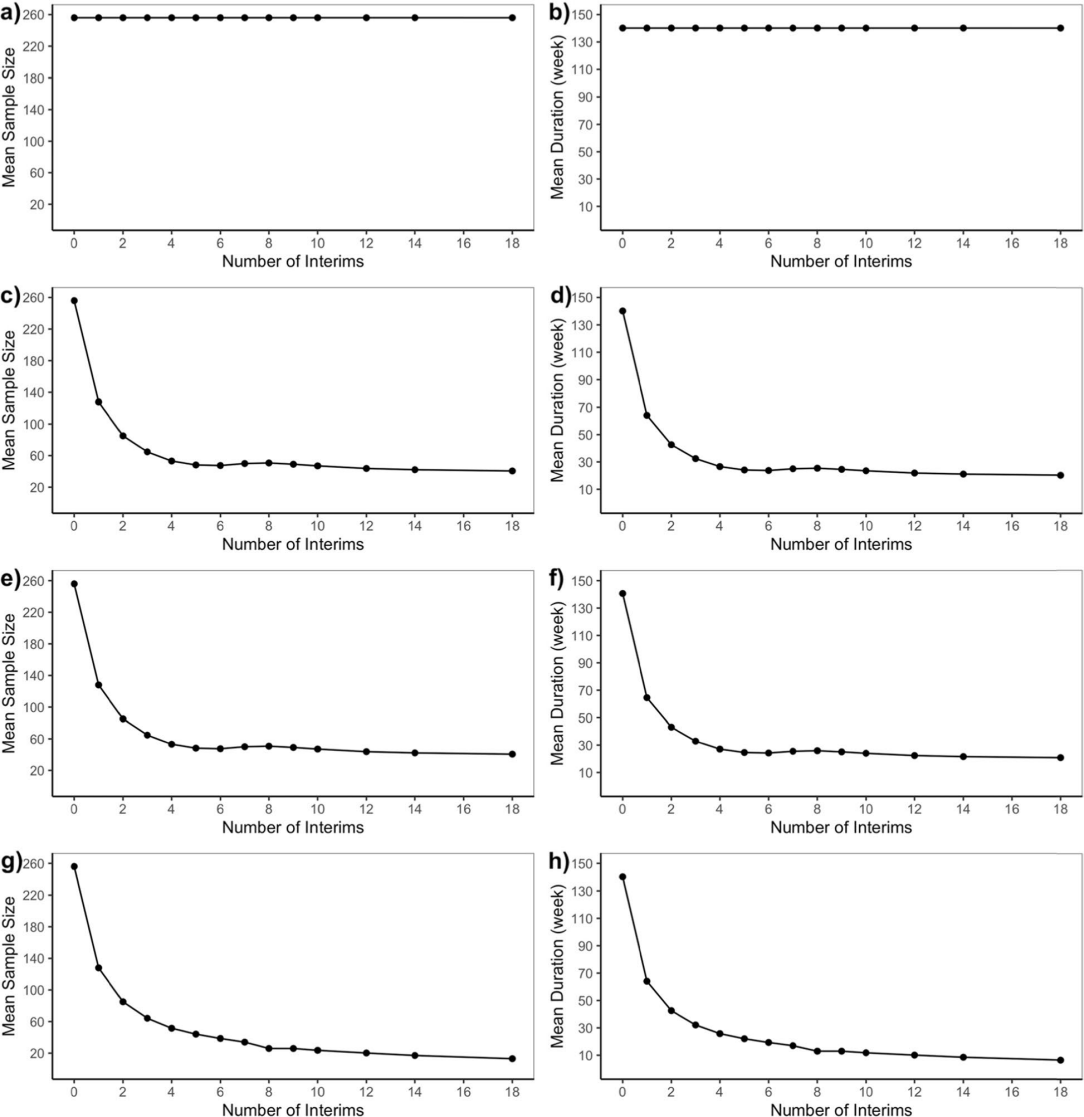
Mean Sample Size and Trial Duration for Bayesian Adaptive Designs under the Harmful Scenario. **a** and (**b**) are Bayesian adaptive design 1 that only allow early stopping for success based on skeptical prior; (**c**) and (**d**) are Bayesian adaptive design 2 that only allow early stopping for futility based on enthusiastic prior; (**e**) and (**f**) are Bayesian adaptive design 3 that allow early stopping for success based on skeptical prior, or early stopping for futility based on enthusiastic prior; (**g**) and (**h**) are Bayesian adaptive design 4 that allow early stopping for either success or futility both based on non-informative prior

The harmful scenario is defined as the case where the true treatment effect for Botox 4 U/kg group is inferior to control with a difference -0.05 (SD = 0.1), i.e., effect size is -0.5. Under the harmful scenario, we evaluated the operating characteristics for Bayesian adaptive designs including rates of early or late success, early or late futility or inconclusive results: all the Bayesian adaptive designs except for design 1 resulted in a 100% early futility stop rate, resulting in a large reduction of the overall sample size regardless of prior choices, which we see clearly demonstrated in Fig. 5. Same as the null or alternative scenario, under the harmful scenario, the fixed design with no interim analysis (number of interim analysis = 0) functions as a reference in each of the four adaptive design candidates.

Figure 5 shows that the expected sample size or trial duration could at least be reduced by half with only one interim analysis or reduced by two-thirds with two interim analyses for all the adaptive designs except Bayesian adaptive design 1. The amount of reduction in expected sample size or trial duration is similar in Bayesian adaptive design 2 and 3, and more aggressive in Bayesian adaptive design 4.

A decision could be made based on simulation results under harmful scenario jointly with the ones under null or alternative scenarios: Bayesian adaptive design 1 does not allow for early futility stopping which clearly risks exposing subjects to ineffective or even harmful treatment effect. While Bayesian adaptive design 4 aggressively minimizes the sample size more than the other designs in the harmful scenario, the sacrifice in power when an increasing number of interim analyses were performed was too great, making this design undesirable overall. Bayesian adaptive designs 2 and 3 fall in-between, with less aggressive futility analyses yielding larger expected sample sizes, while maintaining reasonable statistical power.

### Design justification

Overall, these simulations demonstrate that Bayesian adaptive design 3 (incorporating both skeptical and enthusiastic priors) provides a suitable balance and yields favorable *Operating Characteristics* compared to the alternative designs (incorporating only one type of prior belief or only using either an early success or early futility assessment) even when performing an increasing number of interim analyses.

**Fig. 6:**
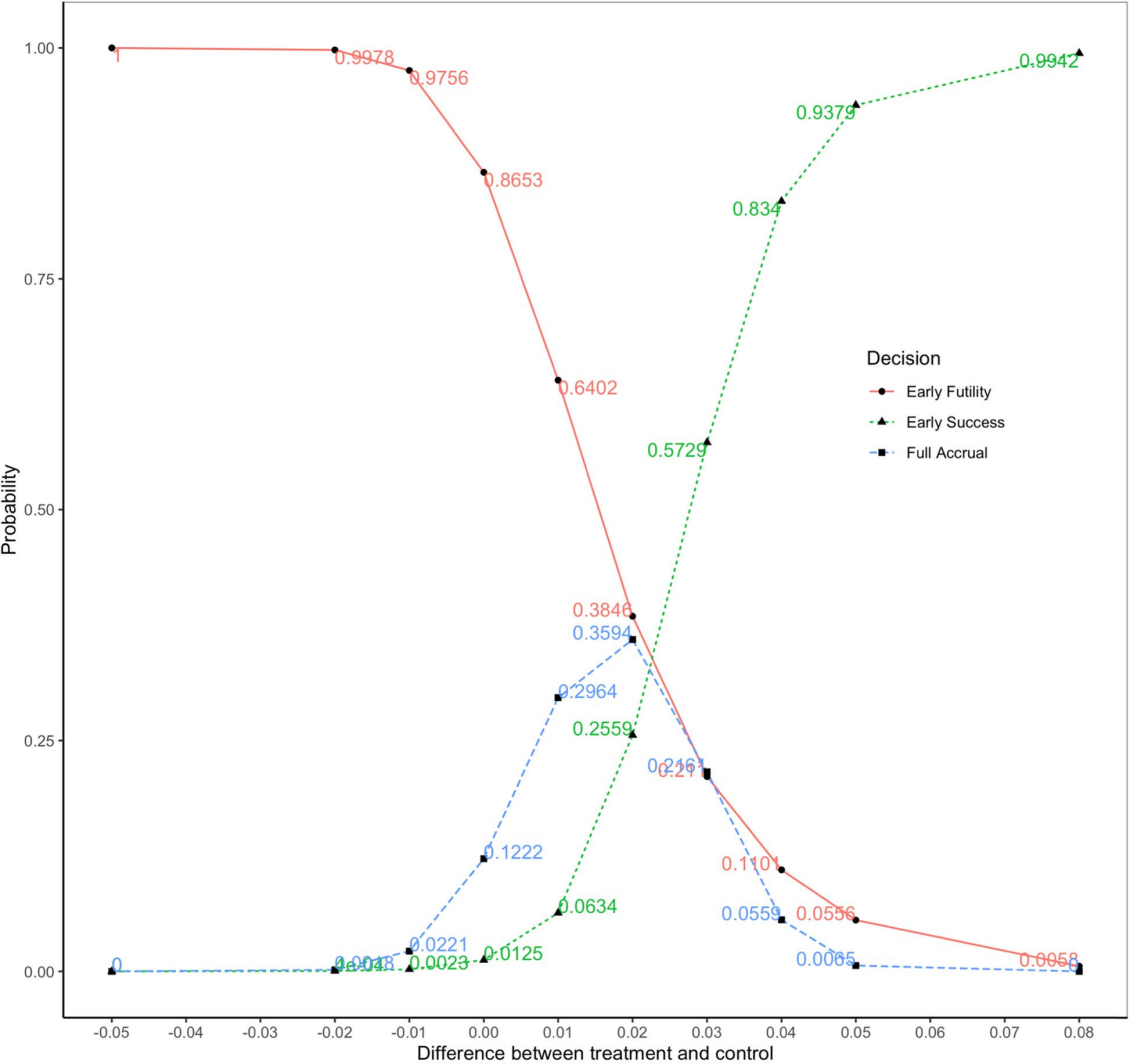
Bayesian Adaptive Design Property the Preferred Design Demonstrating Early Success, Early Futility, and Full Accrual Results

In Fig. 4, we observe that the expected sample size or expected trial duration reduced the most for adaptive design 3 with 6 interim analyses and then produced diminishing returns beyond this point. Figure 6 presents the simulation results for Bayesian adaptive design 3 with 6 evenly spaced interim analyses every 37 subjects. The x-axis is the difference between treatment and control, from -0.05 to 0.08, and the y-axis shows the proportion of the 10,000 simulated trials either stopped early for success or futility or continued to full accrual (late success/late futility/inconclusive).

The green curve is the probability of early stopping for success. The probability of early stopping for success increases with the increase in treatment difference. When the true treatment difference is around 0.05 (i.e., the treatment effect observed in adults), 93.8% of times the trial may be stopped early for success, compared to over 99% for no interim analysis, indicating a slight loss in power for the ability to stop early for success.

The red curve is the probability of stopping early for futility. When the treatment effect is zero or in the harmful direction, from -0.05 to 0, the chance of stopping for futility always exceeds 86%. The probability of early stopping for futility decreases as the treatment difference increases. When the treatment difference is higher than 0.02, the chance that the trial would be stopped for early futility is less than 3%.

The blue curve is the probability of the simulated trials continuing up to full accrual (late success/late futility/inconclusive) without early stopping for either success or futility, whose parabola shape shows that early stopping for either success or futility might be harder to achieve if the true treatment effect seems ambiguous, i.e., we are not sure if it’s harmful or beneficial.

**Fig. 7:**
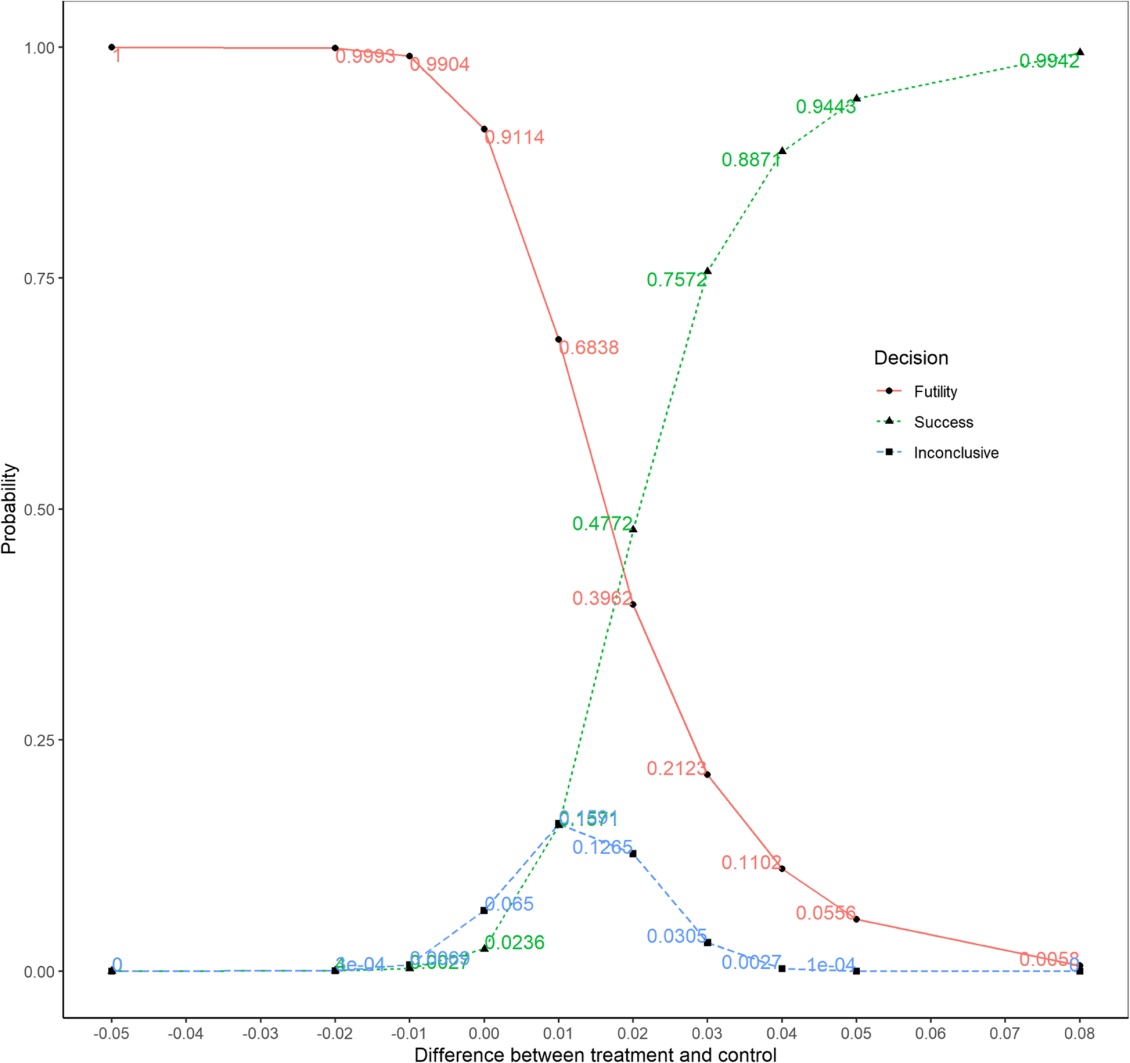
Bayesian Adaptive Design Property for the Preferred Design Demonstrating Success, Futility, and Inconclusive Results

Figure 7 is a variation of Fig. 6, which shows the proportion of the 10,000 simulated trials either achieved success or futility or inconclusive results. The green curve is the probability of achieving success at either interim or final analyses, which increases with the increase in treatment difference. When the true treatment difference is ineffective or harmful, from 0 to -0.05, the chance of concluding the trial was successful is below 2.5%, indicating that type I error rate is well controlled. The red curve is the probability of achieving futility at either interim or final analyses, which decreases with the increase in treatment difference. The blue curve is the probability of the inconclusive trials which did not achieve either success or futility.

## Discussion

### Prior choices

In this paper, we aimed at exploring the flexibility of Bayesian adaptive designs to incorporate different prior beliefs into the clinical trials, which is one of the greatest strengths of the Bayesian methodology. In our re-design for the case study, the enthusiastic prior incorporated in the proposed Bayesian adaptive design for the pediatric clinical trial is based on similar historical adult clinical trial. In practice, to utilize data from adult trials as enthusiastic prior data for pediatric trials, it must first be determined whether it is reasonable to assume that the adult data are relevant to the pediatric patient population. Challenges exist in quantifying the level of relevance of historical adult data. Here one needs to be aware of the risk of overrating the relevance of the adult data, that is if we over rely on the adult data then we will end up needing more patients to demonstrate that the drug is ineffective in pediatrics. The goal is to identify a weight that will prevent early stopping if we have some initial data that is less favorable, without overweighing less favorable pediatric data as we gain additional patients. Modeling & simulation [33] is a useful tool to explore and set expectations on the relevance of the adult data.

When historical adult data are not available, another way to quantify prior information for new pediatric trial is to consider prior elicitation, an approach of combining opinions from different experts in an explicitly model-based way to form a valid subjective prior under the Bayesian framework [34]. For examples of prior elicitation, see Hampson et al. (2015) [35] and Jansen et al. (2020) [36], both utilized the results from an elicitation meeting to create prior probability distributions to assist with the design and planning of a Bayesian trial. Some other studies on prior elicitation considered a mixture of prior beliefs from different clinicians: Gajewski and Mayo (2006) [37] used a mixture of beta priors elicited from clinicians with opposite viewpoints for binomial endpoints in phase II clinical trial, and Moatti et al. (2016) [38] used a mixture of normal priors elicited from experts for log hazard ratio in phase III survival trial. Another standard approach for informative prior incorporation is power prior, which is defined to be the likelihood function based on the historical data raised to a power parameter that enables the historical data to be weighted relative to the current data [39, 40]. The power prior approach has been recently applied in many fields such as clinical trials [41, 42], genetics research [43], environmental studies [44], etc. Later introduced by Hobbs et al. (2011) [45], commensurate prior is an extension of the traditional power prior approach to allow for the commensurability of the information in the historical and current data to determine how much historical information is used, and its applications in prior elicitation have been recently developed and discussed in [24, 46]. Additionally, the elicitation of specific values of the power parameter could also be done via a meta-analytic argument that assumes the historical and current parameter as exchangeable [47, 48]. Schmidli et al. (2014) [49] derived a Bayesian meta-analytic-predictive prior from historical data to be combined with the new data, and demonstrated its applications in clinical trials with historical control information.

Note that prior choices are not limited to the two extreme viewpoints illustrated in this paper and previous literatures. Ye et al. suggested that alternative designs for early phase pediatric clinical trials using noninformative prior instead of skeptical prior for early success criteria could be considered to improve power with a reasonable inflation in false-positive rate [20]. In the rare diseases or when the disease is life-threatening or severely debilitating with an unmet medical need this trade-off may be warranted [35]. We have compared the design property of this alternative design with our proposed innovative design 3 when performing 6 equally spaced interim analyses at every 37 subjects. For our case study, the simulated trial can be stopped early for efficacy or futility at the same probability levels under both designs, therefore the alternative design could not improve power significantly. The possible explanation is that our case study is a phase III trial with much more abundant sample size compared to the early phase study analyzed in Ye et al. [20], so studies with sufficient sample size are more robust to the change in viewpoint from no strong opinion to skeptical when the trial data will dominate the results. We also found when higher number of interim analyses were performed, stricter skeptical prior would be needed to balance operating characteristics including type I error rate and power, which are the main factor for consideration.

Our choice of prior to optimize control of the type I error rate was based solely on simulations. As the number of interim analyses increases the larger the degree of skepticism that is needed to control the type I error at the nominal level of 2.5% and this comes at the cost of decreased power.

### Limitations

In our case study, to account for multiplicity issue and preserve the intended significance level and power, the stopping boundary for early or late success were calibrated to ensure a type I error rate of 2.5% for the one-sided test of treatment superiority to control, while the stopping boundary for early or late futility was determined to ensure early stopping while preserving sufficient power. It is clear that if different values had been chosen for the stopping boundary, different decisions may have been made at the interim analyses. For instance, if the proposed Bayesian adaptive design 3 used less aggressive stopping boundaries for futility, higher power could be obtained, although the study would be more likely to run for longer, exposing patients to ineffective or harmful treatment. Moreover, the Haybittle–Peto boundary considered in this paper is simple to understand, implement, and describe, but often criticized for being too conservative as it only allows early trial stopping for overwhelmingly large difference between the treatments [50]. Other common boundary methods could be further explored to adjust for multiplicity: O'Brien-Fleming method which allows early stopping boundary to vary at every interim look [51], the flexible alpha spending function developed by Lan and DeMets (1983) which does not require the pre-specification of the interim timing [52], etc.

Overall, the community of prior approach demonstrates promise, though will require extended discussion, and thought on the prior choice for pediatric trial designs. Additionally, the community of prior approach incorporating both skeptical and enthusiastic prior could have been compared to other priors (mixture prior, power prior, etc.) in a Bayesian adaptive design setting and we plan to compare them in our future work.

In this paper, we also investigated the impact of an increasing number of interim analyses. An increase in the number of interims would have led to smaller expected sample size and shorter trial duration, but at the cost of increased operational complexity at each interim analysis [53] due to time requirements for data cleaning, performing the analysis and presentations of the results and an overall loss of power. Therefore, we need to be aware of the trade-off between early trial cessation and operational cost.

## Conclusion

In this paper, we have shown through a case study how to innovatively re-design a pediatric phase III trial incorporating a community of prior belief. We also justified the advantage of the innovative adaptive design by comparing it with several alternative adaptive designs only incorporating one kind of prior belief. Simulation results showed that compared to alternative designs, the innovative design offers good control of frequentist operating characteristics including acceptable type I error, sufficient power, fewer patients recruited on average than the original target sample size, and shorter trial duration when performing an increasing number of interim analyses.

In conclusion, the primary benefit of Bayesian adaptive designs is to improve study efficiency, to provide more flexible trial conduct, and to treat more patients with more effective treatments in the trial while maintaining desirable frequentist operating characteristics. This is of particular benefit when accrual to a pediatric clinical trial may be prolonged in the case of cancer and other rare pediatric diseases.

## Supplementary Information

**Additional file 1: Appendix I.** FACTS screen-cuts. **Appendix II.** R code.

**Additional file 2: Appendix III: Table 1.** Operating characteristics for Bayesian adaptive design 1. **Table 2.** Operating characteristics for Bayesian adaptive design 2. **Table 3.** Operating characteristics for Bayesian adaptive design 3 (proposed). **Table 4.** Operating characteristics for Bayesian adaptive design 4. **Table 5.** Operating characteristics for Frequentist group sequential design.

## Data Availability

The data used in this study were generated via simulation. The FACTS screen-cuts for the case study and R code for the additional handling of FACTS simulation output were organized as a step-by-step tutorial, available in the supplementary file 1. The FACTS files are available on reasonable request from the corresponding author (Yu Wang).

## Abbreviations

FDA: Food and Drug Administration
EMA: European Medicines Agency
CI: Credible Interval
SD: Standard Deviation
GSD: Group Sequential Design
