# Supplementary material for: Bayesian adaptive design for pediatric clinical trials incorporating a community of prior beliefs
Source: BMC Med Res Methodol. 2022 Apr 21;22:118. doi: 10.1186/s12874-022-01569-x (PMC9027907; doi:10.1186/s12874-022-01569-x)
Supplement: Supplementary file 1 — Additional file 1: Appendix I. FACTS screen-cuts. Appendix II. R code. [file 12874_2022_1569_MOESM1_ESM.docx]

# Appendix I: FACTS screen-cuts

## Step 1: FACTS design under skeptical prior

Create a FACTS adaptive design with the skeptical prior and include the interims and the QOIs but do not implement any stopping criteria so all interims are evaluated, and every simulation runs to full accrual and final analysis, then output weeks files for every simulation.

Study Information


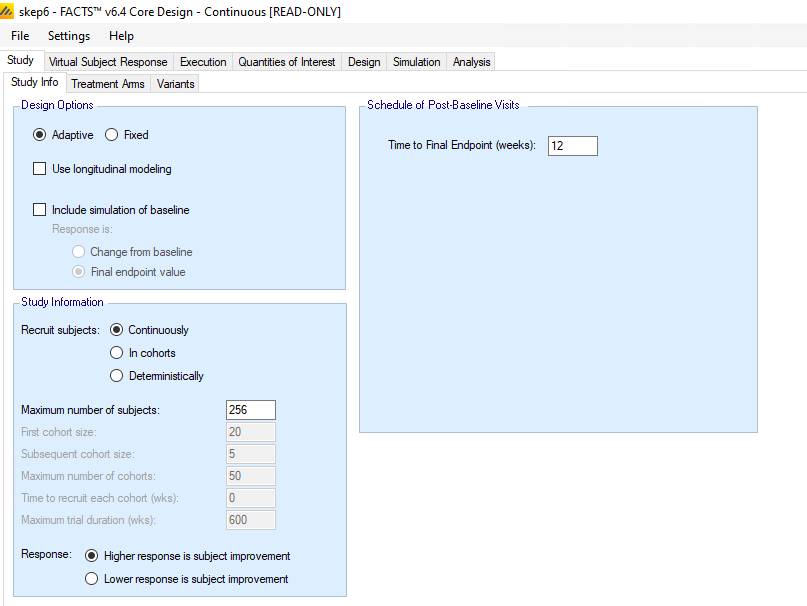


Treatment Arms


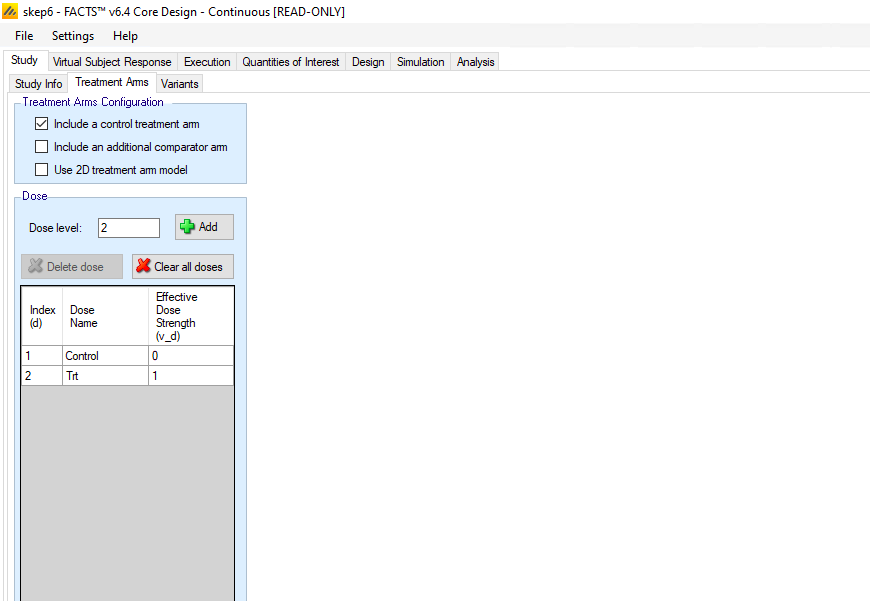


Dose Response under H0 (no difference)


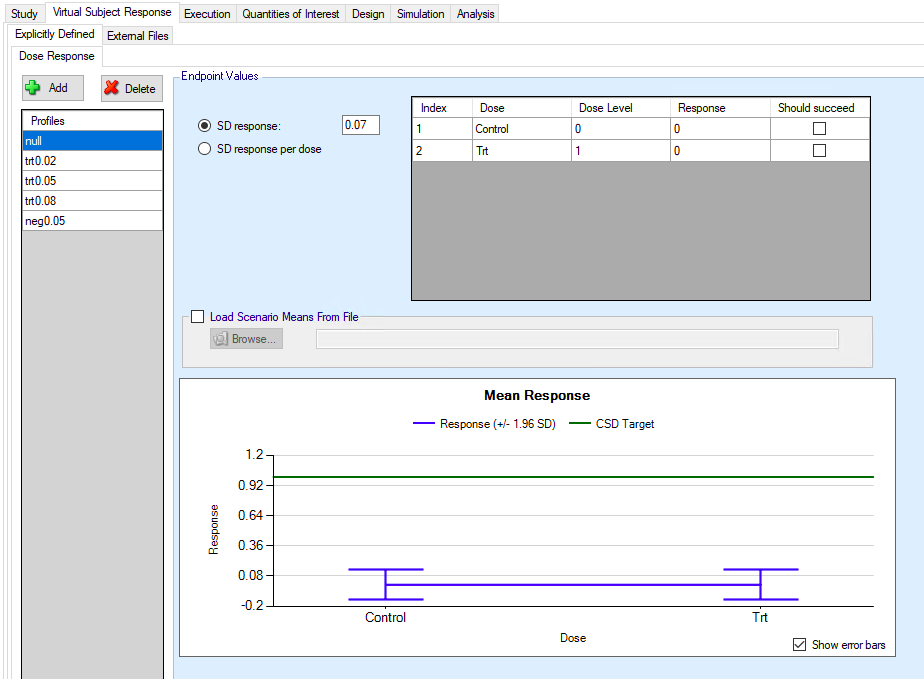


Dose Response under H1 (target difference)


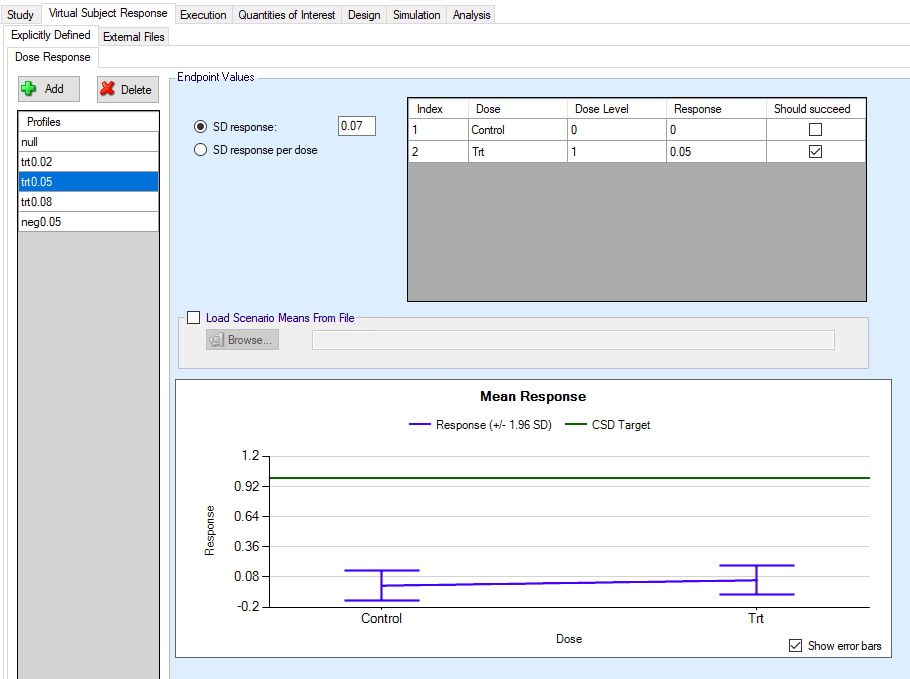


Timing of Interims


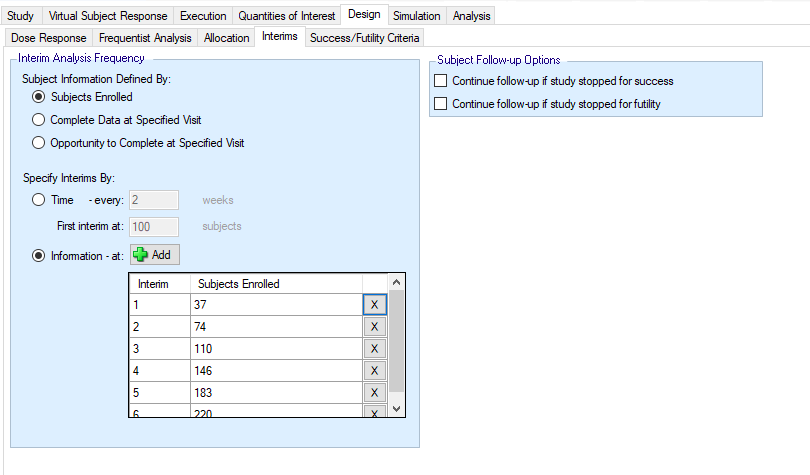


Independent Dose Model under Skeptical Prior


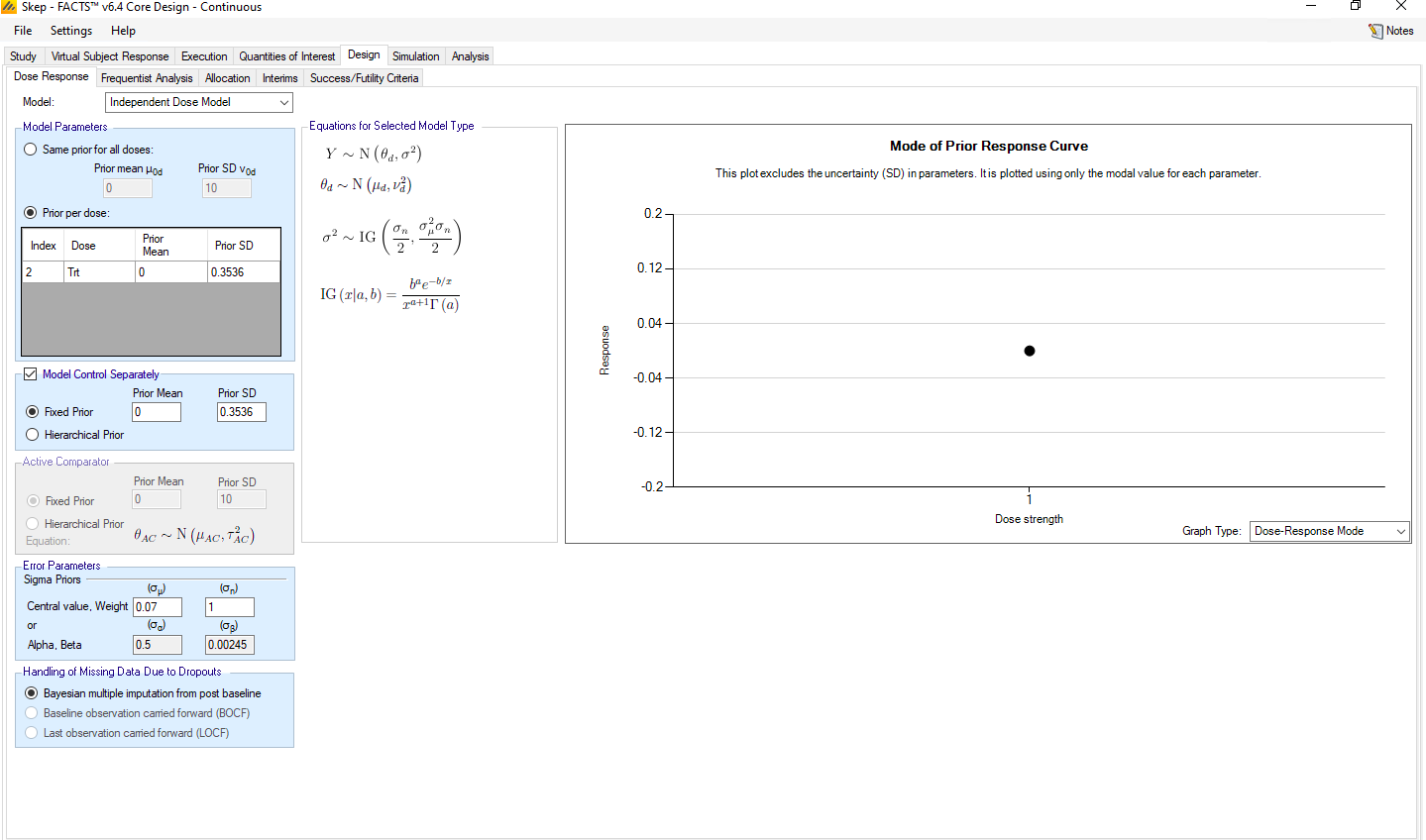


Success Criteria under Skeptical Prior

Success criteria at interim analyses (these criteria will apply at all intervening interims until the next interim for which criteria are defined):


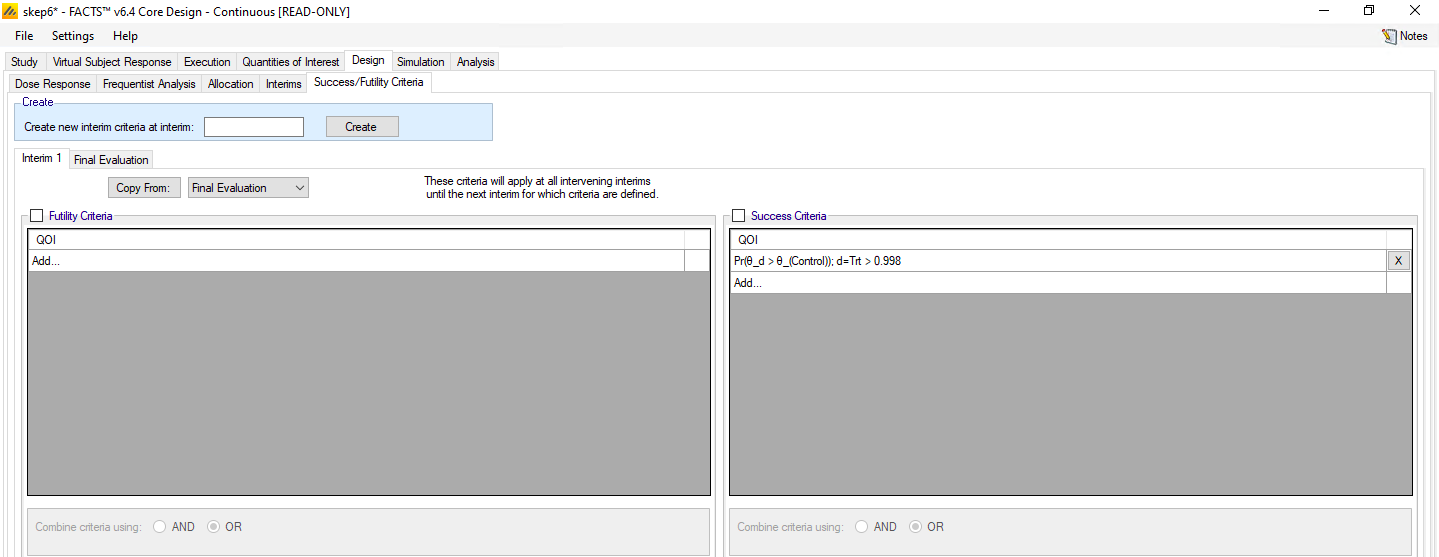


Success criteria at final analysis:
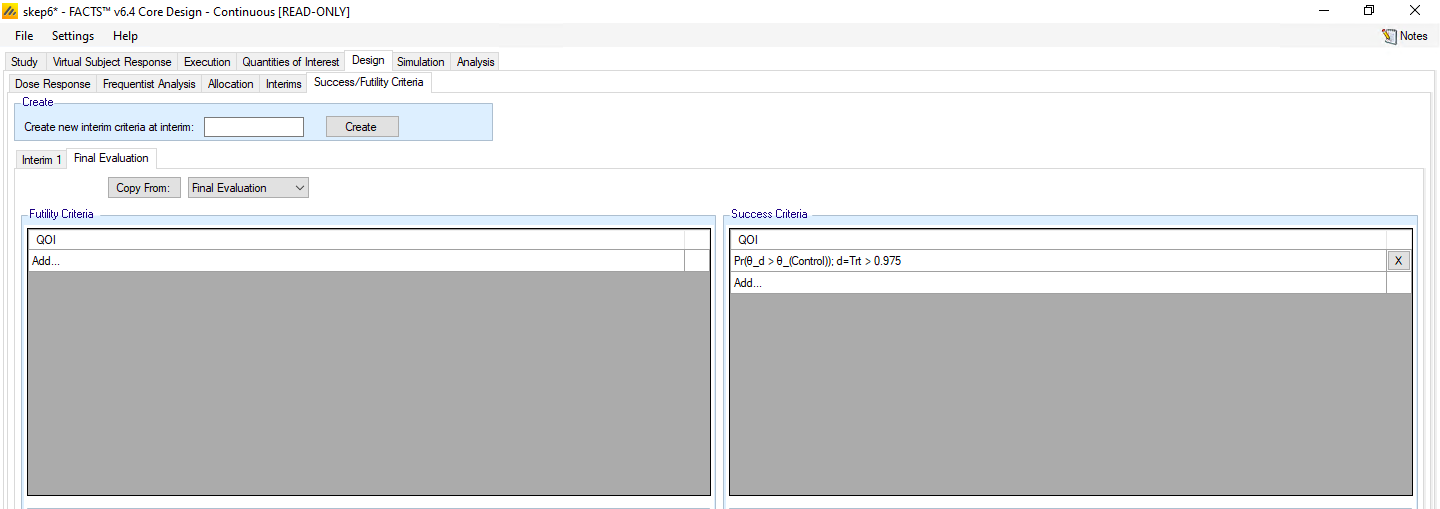


## Step 2: FACTS design under enthusiastic prior

Create a new FACTS adaptive design and change the prior to the enthusiastic prior and re-simulate without adaptation by keeping the same random number seed and making no other changes so that exactly the same patient responses are simulated.

Independent Dose Model under Enthusiastic Prior


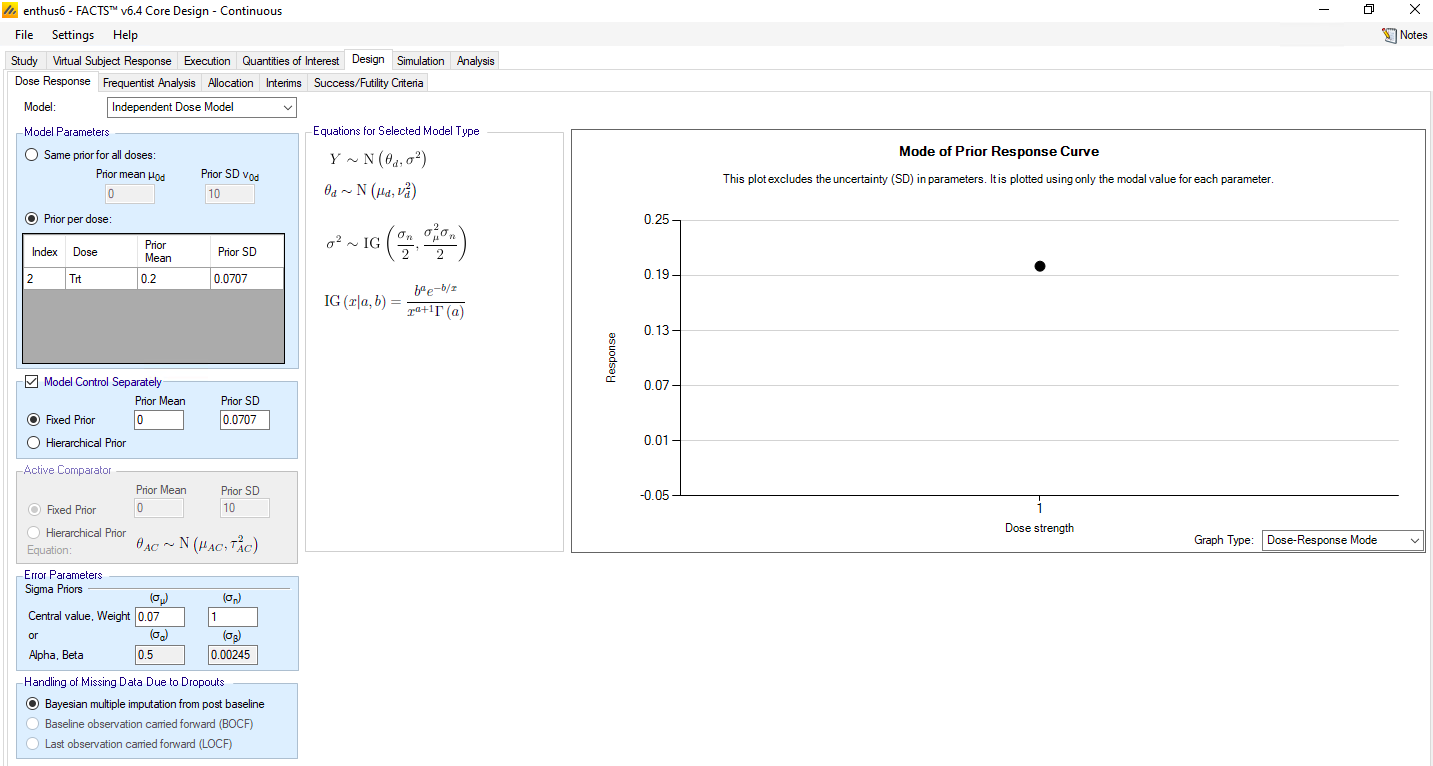


Futility Criteria under Enthusiastic Prior

Futility criteria at interim analyses (these criteria will apply at all intervening interims until the next interim for which criteria are defined):


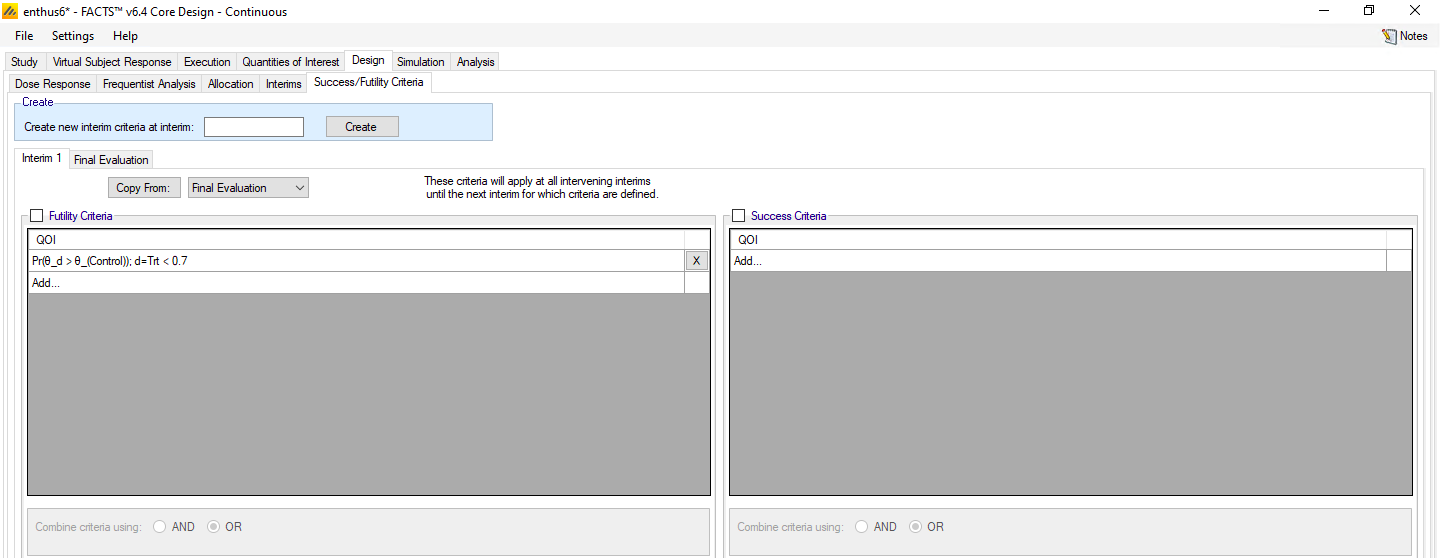


Futility criteria at final analysis:


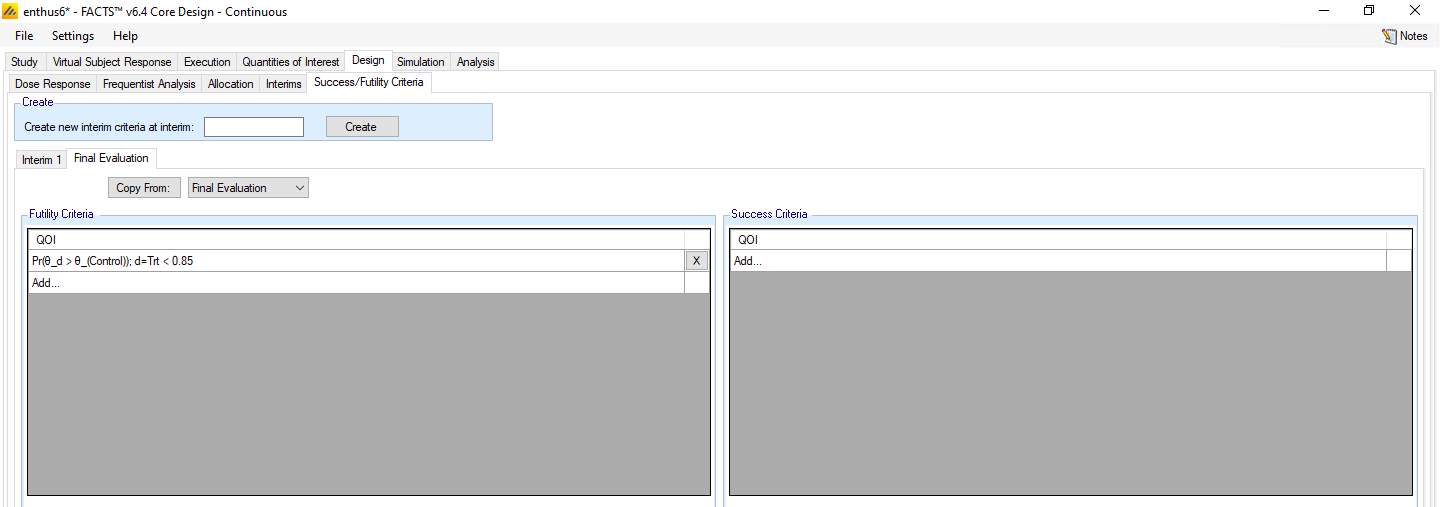


## Step 3: Aggregate the file for each design

Aggregate the weeks files for FACTS designs simulated of the same trials but with skeptical or enthusiastic prior from Step 1 & 2 separately.

Take the FACTS design simulated with skeptical prior from Step 1 for example:


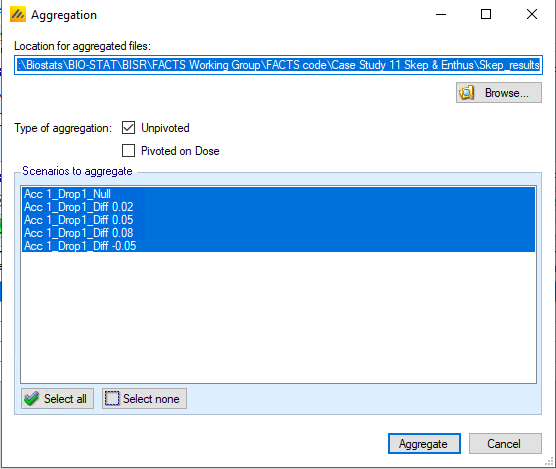


# Appendix II: R code

## Step 4: Merge files in R

Load the 2 sets of aggregated weeks files into R and join them on the Sim and Scenario ID columns so we have posterior probabilities under either skeptical or enthusiastic prior at each interim.

#------- Prepare the datasets ------------

# Aggregated week files

library(readxl)

week_succ_skep_int6_d3 <- read.csv("skep6_results/agg_skep6_weeks.csv", header = T, skip = 2)

week_fut_enthus_int6_d3 <- read.csv("enthus6_results/agg_enthus6_weeks.csv", header = T, skip = 2)

# Merge two separate week datasets on scenario, simulation, interim timing (weeks, # of subjects)

merged_week_int6_d3 <-

merge(week_succ_skep_int6_d3,

week_fut_enthus_int6_d3,

by=c("X.Scenario.ID", "Sim", "InterimNumber", "X.Weeks", "X.Subjects"))

# Generate subsets for each hypothetical scenario

library(tidyverse)

merged_week_int6_d3_null <-

merged_week_int6_d3 %>%

filter(X.Scenario.ID == 1)

merged_week_int6_d3_trt0.02 <-

merged_week_int6_d3 %>%

filter(X.Scenario.ID == 2)

merged_week_int6_d3_trt0.05 <-

merged_week_int6_d3 %>%

filter(X.Scenario.ID == 3)

merged_week_int6_d3_trt0.08 <-

merged_week_int6_d3 %>%

filter(X.Scenario.ID == 4)

merged_week_int6_d3_neg0.05 <-

merged_week_int6_d3 %>%

filter(X.Scenario.ID == 5)

## Step 5: Decision-making in R

Analyze the joined data for each simulated trial to see which stops early for success on the skeptical prior at interims, which stops early for futility on the enthusiastic prior at interims, which makes no early stopping up to full accrual or reach inconclusive at final analysis. The code for each scenario are the same, so take the alternative hypothetical scenario (H1) for an example:

#-------- alternative hypothetical scenario (H1) (target diff. between trt. and ctrl. = 0.05) ------------

# Create flag variables: stop early or late for success or futility

merged_week_int6_d3_trt0.05$early_success <- 0 # 1=stop early for success, 0=don't stop early for success

merged_week_int6_d3_trt0.05$early_futility <- 0 # 1=stop late for futility, 0=don't stop late for futility

merged_week_int6_d3_trt0.05$late_success <- 0 # 1=stop late for success, 0=don't stop late for success

merged_week_int6_d3_trt0.05$late_futility <- 0 # 1=stop late for futility, 0=don't stop late for futility

merged_week_int6_d3_trt0.05$inconclusive <- 0 # 1=inconclusive, 0=conclusive

# Evaluate at each interim & each sim under alternative scenario (trt 0.05)

for (i in c(1:10000)){

cnt2=which(merged_week_int6_d3_trt0.05$Sim == i & merged_week_int6_d3_trt0.05$InterimNumber==999 ) # final look

if (merged_week_int6_d3_trt0.05$Pr.PBO._at_Trt.y[cnt2] < 0.85)

{

# could be modified if the trial stopped early

merged_week_int6_d3_trt0.05$late_futility[cnt2] = 1 # late futility (1=Yes, 0=No)

}

else if (merged_week_int6_d3_trt0.05$Pr.PBO._at_Trt.x[cnt2] > 0.975)

{

# could be modified if the trial stopped early

merged_week_int6_d3_trt0.05$late_success[cnt2] = 1 # late success (1=Yes, 0=No)

}

else {

merged_week_int6_d3_trt0.05$inconclusive[cnt2] = 1 # could be modified if the trial stopped early

}

for (j in 1:6){

cnt=which(merged_week_int6_d3_trt0.05$Sim == i & merged_week_int6_d3_trt0.05$InterimNumber==j ) # interim look

# interim analysis

{

if (merged_week_int6_d3_trt0.05$Pr.PBO._at_Trt.y[cnt] < 0.70)

{

merged_week_int6_d3_trt0.05$early_futility[cnt] = 1

merged_week_int6_d3_trt0.05$late_success[cnt2]= 0

merged_week_int6_d3_trt0.05$late_futility[cnt2]= 0

merged_week_int6_d3_trt0.05$inconclusive[cnt2] = 0

break

break

}

else if (merged_week_int6_d3_trt0.05$Pr.PBO._at_Trt.x[cnt] > 0.998)

{

merged_week_int6_d3_trt0.05$early_success[cnt] = 1

merged_week_int6_d3_trt0.05$late_success[cnt2]= 0

merged_week_int6_d3_trt0.05$late_futility[cnt2]= 0

merged_week_int6_d3_trt0.05$inconclusive[cnt2] = 0

break

break

}

}

}

}

int6_d3_trt0.05 <-

merged_week_int6_d3_trt0.05 %>%

filter(early_success == 1 |

early_futility == 1 |

late_success == 1 |

late_futility == 1 |

inconclusive == 1) %>%

dplyr::select(Sim,

InterimNumber,

early_success,

early_futility,

late_success,

late_futility,

inconclusive,

X.Subjects,

X.Weeks)

int6_d3_trt0.05_table <-

int6_d3_trt0.05 %>%

summarize(early_success = mean(early_success, na.rm = TRUE),

late_success = mean(late_success, na.rm = TRUE),

early_futility = mean(early_futility, na.rm = TRUE),

late_futility = mean(late_futility, na.rm = TRUE),

inconclusive = mean(inconclusive, na.rm = TRUE),

mean_subj = mean(X.Subjects, na.rm = TRUE),

mean_duration = mean(X.Weeks, na.rm = TRUE))
