# Supplementary material for: Bayesian adaptive design for pediatric clinical trials incorporating a community of prior beliefs
Source: BMC Med Res Methodol. 2022 Apr 21;22:118. doi: 10.1186/s12874-022-01569-x (PMC9027907; doi:10.1186/s12874-022-01569-x)
Supplement: Supplementary file 2 — Additional file 2: Appendix III: Table 1. Operating characteristics for Bayesian adaptive design 1. Table 2. Operating characteristics for Bayesian adaptive design 2. Table 3. Operating characteristics for Bayesian adaptive design 3 (proposed). Table 4. Operating characteristics for Bayesian adaptive design 4. Table 5. Operating characteristics for Frequentist group sequential design. [file 12874_2022_1569_MOESM2_ESM.docx]

# Appendix III: Supplementary Tables

**Supplementary Table 1. Operating characteristics for Bayesian adaptive design 1**

| Number of interims | Timing of planned interim | Early stopping decision to be assessed | Type I error rate  (under H0) | Power  (under H1) | Mean sample size  (under H0) | Mean sample size  (under H1) |
| --- | --- | --- | --- | --- | --- | --- |
| 0 | NA (Full accrual 256) | NA | 0.024 | 1 | 256 | 256 |
| 1 | 128 | Success | 0.0256 | 1 | 255.7696 | 161.0624 |
| 2 | 85 170 | Success | 0.0231 | 0.9999 | 255.6314 | 142.856 |
| 3 | 64 128 192 | Success | 0.0279 | 0.9998 | 255.264 | 133.2928 |
| 4 | 51 102 153 204 | Success | 0.0293 | 1 | 255.0338 | 126.4148 |
| 5 | 43 86 129 172 215 | Success | 0.0313 | 1 | 254.7755 | 121.6689 |
| 6 | 37 74 110 146 183 220 | Success | 0.0323 | 1 | 254.7712 | 117.5586 |
| 7 | 32 64 96 128 160 192 224 | Success | 0.029 | 0.9999 | 254.608 | 115.0784 |
| 8 | 28 56 84 112 140 168 196 224 | Success | 0.0301 | 0.9998 | 254.6492 | 112.3624 |
| 9 | 25 50 75 100 125 150 175 200 225 | Success | 0.0343 | 1 | 254.3344 | 110.3256 |
| 10 | 23 46 69 92 115 138 161 184 207 230 | Success | 0.031 | 0.9999 | 254.1313 | 108.6796 |
| 12 | 20 40 60 80 100 120 140 160 180 200 220 240 | Success | 0.0332 | 1 | 254.0564 | 106.6444 |
| 14 | 17 34 51 68 85 102 119 136 153 170 187 204 221 238 | Success | 0.0375 | 1 | 253.6614 | 104.0827 |
| 18 | 13 26 39 52 65 78 91 104 117 130 143 156 169 182 195 208 221 234 | Success | 0.0346 | 0.9999 | 253.6904 | 101.0937 |

**Supplementary Table 2. Operating characteristics for Bayesian adaptive design 2**

| Number of interims | Timing of planned interim | Early stopping decision to be assessed | Type I error rate  (under H0) | Power  (under H1) | Mean sample size  (under H0) | Mean sample size  (under H1) |
| --- | --- | --- | --- | --- | --- | --- |
| 0 | NA (Full accrual 256) | NA | 0.0363 | 1 | 256 | 256 |
| 1 | 128 | Futility | 0.0337 | 0.9997 | 179.2128 | 255.9616 |
| 2 | 85 170 | Futility | 0.0302 | 0.9948 | 145.4194 | 255.1108 |
| 3 | 64 128 192 | Futility | 0.0253 | 0.9835 | 125.728 | 252.8768 |
| 4 | 51 102 153 204 | Futility | 0.0253 | 0.9632 | 113.8618 | 248.5274 |
| 5 | 43 86 129 172 215 | Futility | 0.025 | 0.9482 | 107.3465 | 245.1042 |
| 6 | 37 74 110 146 183 220 | Futility | 0.0235 | 0.9394 | 104.8792 | 242.9872 |
| 7 | 32 64 96 128 160 192 224 | Futility | 0.0241 | 0.94 | 102.6752 | 242.9952 |
| 8 | 28 56 84 112 140 168 196 224 | Futility | 0.0206 | 0.947 | 101.9196 | 244.6944 |
| 9 | 25 50 75 100 125 150 175 200 225 | Futility | 0.0243 | 0.9474 | 100.0182 | 244.9594 |
| 10 | 23 46 69 92 115 138 161 184 207 230 | Futility | 0.0218 | 0.9444 | 97.8897 | 244.3447 |
| 12 | 20 40 60 80 100 120 140 160 180 200 220 240 | Futility | 0.0217 | 0.9345 | 92.6844 | 242.11 |
| 14 | 17 34 51 68 85 102 119 136 153 170 187 204 221 238 | Futility | 0.0246 | 0.9202 | 89.9189 | 238.9066 |
| 18 | 13 26 39 52 65 78 91 104 117 130 143 156 169 182 195 208 221 234 | Futility | 0.0186 | 0.909 | 84.2882 | 236.4233 |

**Supplementary Table 3. Operating characteristics for Bayesian adaptive design 3 (proposed)**

| Number of interims | Timing of planned interim | Early stopping decision to be assessed | Type I error rate  (under H0) | Power  (under H1) | Mean sample size  (under H0) | Mean sample size  (under H1) |
| --- | --- | --- | --- | --- | --- | --- |
| 0 | NA (Full accrual 256) | NA | 0.0240 | 1.0000 | 256 | 256 |
| 1 | 128 | Success or Futility | 0.0241 | 0.9997 | 179.0515 | 161.0145 |
| 2 | 85 170 | Success or Futility | 0.0211 | 0.9948 | 145.0995 | 142.0929 |
| 3 | 64 128 192 | Success or Futility | 0.0217 | 0.9835 | 125.1709 | 131.0659 |
| 4 | 51 102 153 204 | Success or Futility | 0.0230 | 0.9634 | 113.0510 | 121.8009 |
| 5 | 43 86 129 172 215 | Success or Futility | 0.0233 | 0.9482 | 106.4493 | 115.3536 |
| 6 | 37 74 110 146 183 220 | Success or Futility | 0.0211 | 0.9398 | 103.9777 | 110.5793 |
| 7 | 32 64 96 128 160 192 224 | Success or Futility | 0.0219 | 0.9401 | 101.5398 | 108.3372 |
| 8 | 28 56 84 112 140 168 196 224 | Success or Futility | 0.0205 | 0.9470 | 100.7569 | 106.5291 |
| 9 | 25 50 75 100 125 150 175 200 225 | Success or Futility | 0.0258 | 0.9476 | 98.5551 | 104.5224 |
| 10 | 23 46 69 92 115 138 161 184 207 230 | Success or Futility | 0.0236 | 0.9443 | 96.2448 | 102.7225 |
| 12 | 20 40 60 80 100 120 140 160 180 200 220 240 | Success or Futility | 0.0234 | 0.9347 | 91.0867 | 99.7080 |
| 14 | 17 34 51 68 85 102 119 136 153 170 187 204 221 238 | Success or Futility | 0.0280 | 0.9205 | 88.0239 | 96.2275 |
| 18 | 13 26 39 52 65 78 91 104 117 130 143 156 169 182 195 208 221 234 | Success or Futility | 0.0237 | 0.9091 | 82.5455 | 92.1528 |

**Supplementary Table 4. Operating characteristics for Bayesian adaptive design 4**

| Number of interims | Timing of planned interim | Early stopping decision to be assessed | Type I error rate  (under H0) | Power  (under H1) | Mean sample size  (under H0) | Mean sample size  (under H1) |
| --- | --- | --- | --- | --- | --- | --- |
| 0 | NA (Full accrual 256) | NA | 0.0248 | 1 | 256 | 256 |
| 1 | 128 | Success or Futility | 0.0231 | 0.999 | 165.9264 | 161.1136 |
| 2 | 85 170 | Success or Futility | 0.0194 | 0.9851 | 125.3127 | 140.8573 |
| 3 | 64 128 192 | Success or Futility | 0.018 | 0.9544 | 102.5472 | 126.8288 |
| 4 | 51 102 153 204 | Success or Futility | 0.016 | 0.8922 | 85.4356 | 113.5867 |
| 5 | 43 86 129 172 215 | Success or Futility | 0.0158 | 0.8207 | 73.477 | 101.8724 |
| 6 | 37 74 110 146 183 220 | Success or Futility | 0.0143 | 0.7212 | 64.2336 | 89.4713 |
| 7 | 32 64 96 128 160 192 224 | Success or Futility | 0.012 | 0.5566 | 53.0656 | 72.9792 |
| 8 | 28 56 84 112 140 168 196 224 | Success or Futility | 0.0035 | 0.1953 | 32.7468 | 39.7241 |
| 9 | 25 50 75 100 125 150 175 200 225 | Success or Futility | 0.0034 | 0.1953 | 32.68 | 39.6286 |
| 10 | 23 46 69 92 115 138 161 184 207 230 | Success or Futility | 0.0014 | 0.1139 | 27.1675 | 31.5586 |
| 12 | 20 40 60 80 100 120 140 160 180 200 220 240 | Success or Futility | 0.0006 | 0.0367 | 21.3028 | 22.7216 |
| 14 | 17 34 51 68 85 102 119 136 153 170 187 204 221 238 | Success or Futility | 0 | 0.0081 | 17.356 | 17.6851 |
| 18 | 13 26 39 52 65 78 91 104 117 130 143 156 169 182 195 208 221 234 | Success or Futility | 0 | 0.0003 | 13.0104 | 13.0416 |

**Supplementary Table 5. Operating characteristics for Frequentist group sequential design**

| Number of interims | Timing of planned interim | Early stopping decision to be assessed | Type I error rate  (under H0) | Power  (under H1) | Mean sample size  (under H0) | Mean sample size  (under H1) |
| --- | --- | --- | --- | --- | --- | --- |
| 0 | NA (Full accrual 256) | NA | 0.0237 | 0.9999 | 256 | 256 |
| 1 | 128 | Success or Futility | 0.1463 | 0.9949 | 128.1792 | 223.5776 |
| 2 | 85 170 | Success or Futility | 0.0176 | 0.9847 | 125.5706 | 139.2512 |
| 3 | 64 128 192 | Success or Futility | 0.0174 | 0.9536 | 102.6944 | 126.112 |
| 4 | 51 102 153 204 | Success or Futility | 0.0151 | 0.8928 | 85.7129 | 113.6779 |
| 5 | 43 86 129 172 215 | Success or Futility | 0.0146 | 0.8219 | 73.4373 | 101.6416 |
| 6 | 37 74 110 146 183 220 | Success or Futility | 0.0139 | 0.7189 | 64.2009 | 89.1733 |
| 7 | 32 64 96 128 160 192 224 | Success or Futility | 0.0114 | 0.5468 | 52.6656 | 72.208 |
| 8 | 28 56 84 112 140 168 196 224 | Success or Futility | 0.0034 | 0.1735 | 31.9741 | 38.1373 |
| 9 | 25 50 75 100 125 150 175 200 225 | Success or Futility | 0.003 | 0.1735 | 31.9179 | 38.0511 |
| 10 | 23 46 69 92 115 138 161 184 207 230 | Success or Futility | 0.0015 | 0.0965 | 26.5705 | 30.266 |
| 12 | 20 40 60 80 100 120 140 160 180 200 220 240 | Success or Futility | 0.0004 | 0.0306 | 21.1572 | 22.3292 |
| 14 | 17 34 51 68 85 102 119 136 153 170 187 204 221 238 | Success or Futility | 0.0001 | 0.0058 | 17.2113 | 17.488 |
| 18 | 13 26 39 52 65 78 91 104 117 130 143 156 169 182 195 208 221 234 | Success or Futility | 0 | 0.0001 | 13.0039 | 13.0117 |
